# Identifying Aspects of the Post-Transcriptional Program Governing the Proteome of the Green Alga *Micromonas pusilla*

**DOI:** 10.1371/journal.pone.0155839

**Published:** 2016-07-19

**Authors:** Peter H. Waltman, Jian Guo, Emily Nahas Reistetter, Samuel Purvine, Charles K. Ansong, Marijke J. van Baren, Chee-Hong Wong, Chia-Lin Wei, Richard D. Smith, Stephen J. Callister, Joshua M. Stuart, Alexandra Z. Worden

**Affiliations:** 1 University of California at Santa Cruz, Baskin School of Engineering, Santa Cruz, California, 95064, United States of America; 2 Monterey Bay Aquarium Research Institute, Moss Landing, California, United States of America; 3 Biological Sciences Division, Pacific Northwest National Laboratory, Richland, Washington, 99352, United States of America; 4 U.S. Department of Energy (DOE) Joint Genome Institute (JGI), Walnut Creek, California, 94598, United States of America; 5 University of California Santa Cruz, Department of Ocean Sciences, Santa Cruz, California, 95064, United States of America; 6 Integrated Microbial Biodiversity Program, Canadian Institute for Advanced Research, Toronto, Canada, M5G 1Z8; Queen's University Belfast, UNITED KINGDOM

## Abstract

*Micromonas* is a unicellular motile alga within the Prasinophyceae, a green algal group that is related to land plants. This picoeukaryote (<2 μm diameter) is widespread in the marine environment but is not well understood at the cellular level. Here, we examine shifts in mRNA and protein expression over the course of the day-night cycle using triplicated mid-exponential, nutrient replete cultures of *Micromonas pusilla* CCMP1545. Samples were collected at key transition points during the diel cycle for evaluation using high-throughput LC-MS proteomics. In conjunction, matched mRNA samples from the same time points were sequenced using pair-ended directional Illumina RNA-Seq to investigate the dynamics and relationship between the mRNA and protein expression programs of *M*. *pusilla*. Similar to a prior study of the marine cyanobacterium *Prochlorococcus*, we found significant divergence in the mRNA and proteomics expression dynamics in response to the light:dark cycle. Additionally, expressional responses of genes and the proteins they encoded could also be variable within the same metabolic pathway, such as we observed in the oxygenic photosynthesis pathway. A regression framework was used to predict protein levels from both mRNA expression and gene-specific sequence-based features. Several features in the genome sequence were found to influence protein abundance including codon usage as well as 3’ UTR length and structure. Collectively, our studies provide insights into the regulation of the proteome over a diel cycle as well as the relationships between transcriptional and translational programs in the widespread marine green alga *Micromonas*.

## Introduction

*Micromonas* is a unicellular green alga that belongs to the prasinophytes, a widespread group of marine phytoplankton that retain characteristics of the algal ancestor of land plants [1, 2]. Together with chlorophyte algae (e.g., *Chlamydomonas reinhardtii*), the prasinophytes sister the streptophytes (land plants), collectively forming the Viridiplantae [3]. Marine ecosystems where *Micromonas* resides undergo constant environmental change through seasonal cycles and more recently anthropogenic influences [4, 5]. Yet the capacity to model how such changes influence growth and CO_2_ uptake by marine algae is hampered by limited understanding of basic cellular processes. Two major impediments to our understanding are that i) the influence of the day-night cycle on protein expression has been characterized in only a few taxa, and ii) the temporal and regulatory relationship between transcriptional and translational expression is not understood. Not only do the stages of gene expression define the most basic aspects of cell physiology, but the interpretation of oceanographic field results relies on understanding the dynamics of gene expression over a diel cycle. Moreover, many field studies rely solely on mRNA expression (metatranscriptomics) to infer protein expression because this data is easier to obtain than global proteomic information.

Factors that affect cellular protein abundance also remain ill-characterized in model organisms. Such factors include mRNA abundance and stability as well as post-transcriptional modifications, localization, amino acid concentration, degradation signaling and translational efficiency. The effect of these post-transcriptional factors on protein expression is often overlooked and their importance debated [6, 7]. An emerging consensus is that mRNA and protein expression generally lack mutual correlation [8–15]. Several reports conclude that mRNA expression alone explains only approximately 40% of the variance observed in protein expression data. Notable exceptions exist and other studies find greater correlations that explain up to 81% of the variance [6, 16, 17]. Computational models have been developed to take into account mechanisms of post-transcriptional control in order to examine the relationship between mRNA and protein expression more deeply. These models broadly follow two distinct approaches, employing either regression-based methodologies [12, 13, 15] or dynamical systems of related-rates [8, 11, 16, 17]. Both approaches incorporate mechanisms to model non-transcriptional factors such as translation as well as mRNA and protein degradation rates.

Most analyses that compare mRNA and protein expression have been limited to analyzing either a single steady-state experimental condition or a single sample at each time point in medically or agriculturally relevant model taxa, e.g., [8, 12, 13, 15]. A number of these studies generated protein data, but the corresponding mRNA data came from public resources and therefore different samples. Several more recent reports [18, 19] have utilized matched mRNA and proteomic data from multiple samples that were used to either examine differences between specific cell types [18] or variation between individuals [19]. Studies that have performed longitudinal, time-series analyses have generally examined toxic or environmental responses in which the organism was required to quickly adapt or respond to an environmental cue or stimuli [16, 20, 21]. For marine algae, paired samples for mRNA and protein analysis were used to investigate response of the marine cyanobacterium *Prochlorococcus* to the diel cycle, a key environmental stimulus in nature [22]. An advantage was that the majority of cells in the sampled population were in the same cell-cycle stage due to strong synchronization by the light:dark cycle. Waldbauer et al. observed a significant divergence between the mRNA and protein expression responses to the diel for the 312 detectable proteins that were studied. Although these 312 proteins represent only 16% of the predicted protein-coding genes in *Prochlorococcus*, the findings have important implications for interpretation of transcriptome and meta-transcriptome studies. This type of study is essential for interpreting dynamics in nature where protein measurements, and even mRNA expression data, can be difficult to obtain and are used to infer environmental factors that control growth and shape microbial community structure. Diversity within the microbial world is broad and apart from the studies of Waldbauer et al. on *Prochlorococcus*, relationships between mRNA and protein expression programs have not been systematically studied in ecologically important marine algae.

We present a joint mRNA and protein analysis of the eukaryotic alga *Micromonas pusilla* over the course of the diel cycle. *Micromonas* is a broadly distributed picoeukaryotic genus (<2 μm diameter) that has reportedly increased in the Canadian Arctic in association with climate-induced changes [5]. Here, *M*. *pusilla* (CCMP1545) cells were synchronized by the diel cycle and used as a single source for mRNA and proteomic characterization at four time points. In addition to investigating global proteomic changes, we developed computational models to characterize the relationship between expression programs. Estimates of the rates of translation, transcription and degradation are not available for *Micromonas*. Therefore, we used a regression-based approach to analyze the matched RNA-Seq and liquid-chromatography mass-spectrometry (LC-MS) -based proteomics data, with the goal of identifying putative mechanisms of post-transcriptional control within the organism. Finally, we sought to develop a model that used mRNA sequence features to help account for changes in protein abundance.

## Materials and Methods

### Culturing and cell harvests

*M*. *pusilla* CCMP1545 was maintained in light acclimated (14:10 light dark cycle), mid-exponential growth (semi-continuous batch culture) for >10 generations before the experiment. Cultures were monitored by flow cytometry each morning. The 24 hr experiment was performed in biological triplicate under the same conditions as for culture acclimation, specifically, 220 μE m^-2^ s^-1^ photosynthetically active radiation (PAR) in L1 [23] media at 18°C. A day prior to the 24 hr experiment, cultures were transferred into multiple polycarbonate, vented tissue culture flasks (as also used for acclimation phase) of 500 mL with 300 mL culture and flasks were sacrificed at each of the four time points. Axenicity was verified using DAPI staining and epifluorescence microscopy as well as inoculation into a bacterial test medium throughout the experiments [24]. For proteomic analyses cells were centrifuged at 10,000 x *g* for 12 min and after supernatant removal the remaining material was aliquoted and centrifuged in 2 ml volumes at 8,000 x *g* for 10 min, the supernatant again removed, and pellets immediately frozen in liquid nitrogen. For RNA-Seq cells were harvested by filtration as detailed in [3].

### Proteomics

Proteins from 1.6 x 10^8^ to 2.7 x 10^8^ pelleted CCMP1545 cells were extracted from whole cell (global), soluble, and insoluble lysate fractions. The pelleted cells were washed and suspended in 100 mM NH_4_HCO_3_ (pH 8.4), then lysed via Pressure Cycling Technology (PCT) using a Barocylcer (Pressure BioSciences Inc., South Easton, MA) as previously described [25]. Proteins were then isolated from other cellular components and quantitated for bulk protein concentration (BCA assay). Proteins were denatured by adding Urea to a final concentration of 8 M, and reduced through addition of fresh dithiotreitol (DTT) to reach a final concentration 5 mM; the solution was incubated at 60°C for 30 min. Following incubation, a volume of 0.5 M Iodoacetmide (IAM) was added to each fraction (alkylation step) to obtain a final concentration of 40 mM, then incubated for 1 hr 37°C (protected from light). Proteins were digested using sequencing-grade trypsin (Roche, Indianapolis, IN) at a unit-to-protein ratio of 1:50, and the resulting peptides were desalted by using a strong cation-exchange (SCX), C-18 SPE column (Supelco, St. Louis, MO) following established protocols [26]. A total of 12 samples were analyzed by a Velos Orbitrap mass spectrometer (Thermo Fisher Scientific, San Jose CA) coupled to an online reverse phase HPLC separation. Instrument operating conditions and HPLC conditions have been previously described [27]. For each sample, four technical replicates (in some cases 5) high resolution MS and concurrent low resolution MS/MS (CID fragmentation) spectra were generated.

MS/MS spectra were searched via SEQUEST (Ver27, rev 12) with a nominal fragment ion tolerance of ± 0.5m/z against the predicted proteome of *M*. *pusilla* [1] (see also data deposition section). Results were imported into an Accurate Mass and Time (AMT) tag SQL database and filtered to ~1% FDR at the peptide level (peptides having a MSGF spectral probability score of < 1x10^-10^ [28]), providing a look-up database of mass and elution time values for peptides identified in the prospective samples. High resolution MS scans from the same technical replicates were deisotoped using Decon2LS [29] to provide neutral mass and elution time values for all isotopic features in the technical replicates. Isotopic features were combined across MS scans to provide LC-MS elution features for comparison back to the look-up table derived from MS/MS identifications using VIPER [30].

For presence versus absence analysis, proteins represented by a single peptide were first removed. Then, to add confidence to the presence of a protein within a sampled time point, we required unique peptides for a protein to be measured in at least 50% of all instrument datasets (12 to 14 total datasets) generated from technical and biological replicates; thus, low-occurrence peptides were removed from further analysis. In addition, only peptides unique to a given protein were used for abundance analysis.

For abundance analysis, peptide abundances were calculated from the integration of ion intensities (ion current) measured across instrument scans, then log-transformed (base 10). Next, as MS data can often exhibit high variance per peptide, even between technical replicates from the same biological replicate, peptides were filtered by variance, using the “model-based” filtering option from DanteR [31]. Replicate reproducibility of peptide abundances (Figs A-D in S1 File) was evaluated using pairwise Pearson’s Correlation procedure available in DanteR. Following this, proteins represented by a single peptide were also removed. The resulting data set was then quantile normalized [32], after which the replicate samples (both technical and biological samples) were averaged together to provide sample-specific abundance estimates of the peptides, and the peptide-level abundances were summarized to provide protein-level abundance estimates using RRollup method (available in DanteR), using the default options.

#### RNA-Seq

*M*. *pusilla* CCMP1545 polyA RNA was isolated and prepared for sequencing on the Illumina HighSeq platform as described in [3]. Paired end reads were 150 bp each and between 24,491,192 and 67,274,037 reads were attained per sample (average 48,133,473 per sample). The reads were aligned to the CCMP1545 genome using Tophat version 1.4.0 [33]. Aligned reads and evidence-based gene predictions were then used as input to Cufflinks version 2.0.2 [34] with parameters—library-type fr-firststrand—max-intron-length 10000—min-intron-length 20—min-frags-per-transfrag 10—upper-quartile-norm—max-multiread-fraction 1.0—max-bundle-frags 3000000—overlap-radius 1 -v.

#### Functional annotation and pathway identification

Interproscan v5 [35, 36] was used with default settings and including the PANTHER protein set [37, 38] to functionally annotate the predicted proteins of CCMP1545. If a Tigrfam or Panther match was found, its description is used to annotate the protein, giving preference to Tigrfam. Otherwise, the descriptors of any remaining hits were concatenated. Gene ontology (GO) [39] and enzyme commission (EC) [40] identifiers were extracted from all Interproscan results for a given protein. While publicly available pathway annotations for *M*. *pusilla* are available from the Kyoto Encyclopedia of Genes and Genomes [41, 42], we had access to an improved set of *M*. *pusilla* gene models available at JGI and therefore used the PathoLogic tool from the Pathway Tools software suite [43, 44] to infer identify a total of 270 pathways, involving nearly 1800 metabolic reactions and 2000 genes (Table A in S1 File).

#### Determining mRNA and proteomics correlation classes and concordance analyses

To determine correlation classes, mRNA and protein expression profiles for each gene were standardized by converting to z-scores (M = 0, SD = 1) independently for each data type. A gene’s mRNA and protein expression was considered to be highly correlated if they exhibited a Pearson’s correlation coefficient (R) > 0.75. Similarly, mRNA and protein expression profiles were defined to be highly anti-correlation if they exhibited R < -0.75. This also applied when determining whether the expression profiles were delayed by 1 or more time points. A threshold of R > 0.1 was used to determine low correlation. Significance was determined using permutation testing in which we permuted each gene in the high-confidence gene set such that the mRNA and protein expression profiles were randomized. Using this permuted joint matrix, the definitions above were used to determine the total number of genes belonging to each class. 10000 permutations were performed to evaluate the distribution of class memberships and assess significance.

Gene Set Enrichment Analysis (GSEA) was used to identify differential activity at a gene set (or ‘pathway’) level. For each molecular expression type (mRNA and protein), the pairwise Pearson correlation for all pairs of genes in the high-confidence gene set was calculated by determining the Pearson correlation for their expression profiles over the course of the experiment. To make the pathways from this inference compatible with the gene-pair correlations, pathway sets were converted to contain all possible pairings (gene pairs) between the genes of each respective pathway. GSEA was performed for each expression type using these pathway gene-pair sets and the respective gene-pair expression correlations to determine the enrichment score (ES) for each pathway. From these analyses, the ES for each pathway for the mRNA and protein data was calculated and compared for concordance between the two data types. As the range of the ES metric is between -1 and 1, inclusive, the following concordance score was constructed to gauge the degree of concordance (CS) between the mRNA and protein expression data for pathway p:

$${CS}_{p}=sign\left({ES}_{p}^{mRNA}\right)\times sign\left({ES}_{p}^{prot}\right)\times \sqrt{\left|{ES}_{p}^{mRNA}\right|\times \left|{ES}_{p}^{prot}\right|}$$

Where ${ES}_{p}^{mRNA}$ is the enrichment score for pathway p, using the mRNA correlations; while ${ES}_{p}^{prot}$ is the enrichments score of the correlations in the protein data. All GSEA analysis was performed using a Java-based application, available from [45].

#### Clustering, feature collection, partial correlations, MARS and GLM analyses

Tools adapted from the Galaxy Bioinformatics Workflow Project [46, 47] were used for consensus clustering (CCPLUS) [48]. PAM [49] was used as the base learner and the Euclidean distance metric was used to generate the consensus matrix, which was then clustered using HAC, and average linkage. Prior to clustering, the merged expression matrix was constructed by z-transforming (M: 0; SD: 1) the rows (genes) of each molecular expression matrix independently. Following z-transformation, the two expression matrices were concatenated. All enrichment testing was performed using a hypergeometric function, with a Bonferroni corrected significance threshold of 0.05 [50]. Computationally-derived features (Table B in S1 File) were calculated with publicly available tools and features that have been investigated in other taxa [13, 21]. Localization predictions from TargetP [51] and Introner Element status (binary classification on whether a gene’s intron(s) contain an Introner Element [1] were also included. Partial correlations were calculated with the ppcor package (Comprehensive R Archive Network CRAN) [52]. Statistical significance of the correlations and partial correlations was estimated by bootstrap re-shuffling (n = 10000), where mRNA-protein abundance tuples were maintained, while shuffling the gene labels of the expression data types.

Both MARS and generalized linear modelling (GLM) were performed using the earth package, available from CRAN [53]. MARS was performed using the default settings, using generalized cross validation to estimate the final model; while GLM’s were fit using a Gaussian distribution and an identity link function. We also generated GLM and MARS models constrained to only consider mRNA level and the CAI metric, but the performance of these constrained models was considerably worse than we observed for the unconstrained MARS models (maximum R^2^: 0.5; Figs E and F in S1 File). Similarly, when using mRNA alone (Figs G and H in S1 File), no model achieved an R^2^ greater than 0.35 for any sample. These constrained models captured at most 50% of the total variance, indicating that the other sequence features we considered provide significant explanatory power for the protein abundances that were observed.

#### Identification and assessment of HPTR genes

We identified genes whose abundance we hypothesized may be post-transcriptionally regulated (HPTR). We examined sequence features for those with obvious differences between the HPTR and non-HPTR genes. However, Wilcoxon testing failed to identify any features with significantly different distributions between the HPTR genes and non- HPTR genes. To determine whether there were combinatorial effects between the sequence features that separated HPTR genes from non- HPTR genes, we also generated linear classifiers using an elastic net regression [54]; but bootstrap testing indicated these classifiers were not statistically significant. We next generated new MARS models for HPTR genes that were either under-estimated or over-estimated by the previous MARS models to determine whether these were under the influence of a) alternate factors, or b) similar factors as the non-HPTR genes, but to a different degree (as would be indicated by different coefficients of these gene set-specific MARS models). Model accuracies increased for these genes, but bootstrap testing indicated they were not significant.

#### Classification tasks

All classification tasks were performed using linear models regularized with elastic net regression using the GLMNET package [54], available from CRAN, using a binomial family, and the defaults for the remaining parameters (e.g. 10 folds, and the default loss measure, deviance). Classifier testing was also performed using regression trees, naïve Bayesian, and support vector classifiers via the RWeka CRAN package [55], but these were outperformed by GLMNET.

While generating the HPTR gene classifiers, we partitioned the data into 3 sets: non-HPTR genes, HPTR genes whose abundance was over-estimated by the regression models (HPTR+), and those that were under-estimated by models (HPTR-). As such, 2 classifiers were generated, with one distinguishing between the non-HPTR genes and the HPTR+ genes, and similarly, another classifier to distinguish between the non-HPTR and the HPTR- genes. In each case, set membership was balanced using convex pseudo-data (CSD) [56]. Briefly, two samples were selected at random from a smaller class of samples, and a pseudo-sample generated that lies along the convex hull between those samples.

To evaluate the accuracy of the binary classifiers that were generated, we used the balanced success rate metric [57], defined as:

$$BSR=\frac{{SR}_{1}+{SR}_{2}}{F2}$$

Where SR_1_ and SR_2_ are the success rates for classes 1 and 2. The success rate is defined as the fraction of true positives (TP) correctly classified, i.e. *SR* = *TP*/(*TP* + *FN*).

### Data deposition

All LC-MS data have been deposited in the MASSive proteomics data repository (http://massive.ucsd.edu/ProteoSAFe/static/massive.jsp). The accession number is MSV000079604 (ProteomeXchange ID PXD004136). Also included are the instrument data files used to build the AMT tag database (look up table), plus the data files used by VIPER that performs the LC-MS feature finding and database matching (peak matching). The genomic databases (forward and decoy) used for interpreting proteomic data from *Micromonas pusilla* CCMP1545 is available at: http://www.mbari.org/resources-worden-lab/. This genomic database contains the translated protein sequences (forward), and an appended set of sequences that are exactly reversed (decoy), which provided a “noise” pool to allow us to detect false events. RNA-seq data has been deposited in the Short Read Archive under BioProject PRJNA309330.

## Results and Discussion

**Fig 1:**
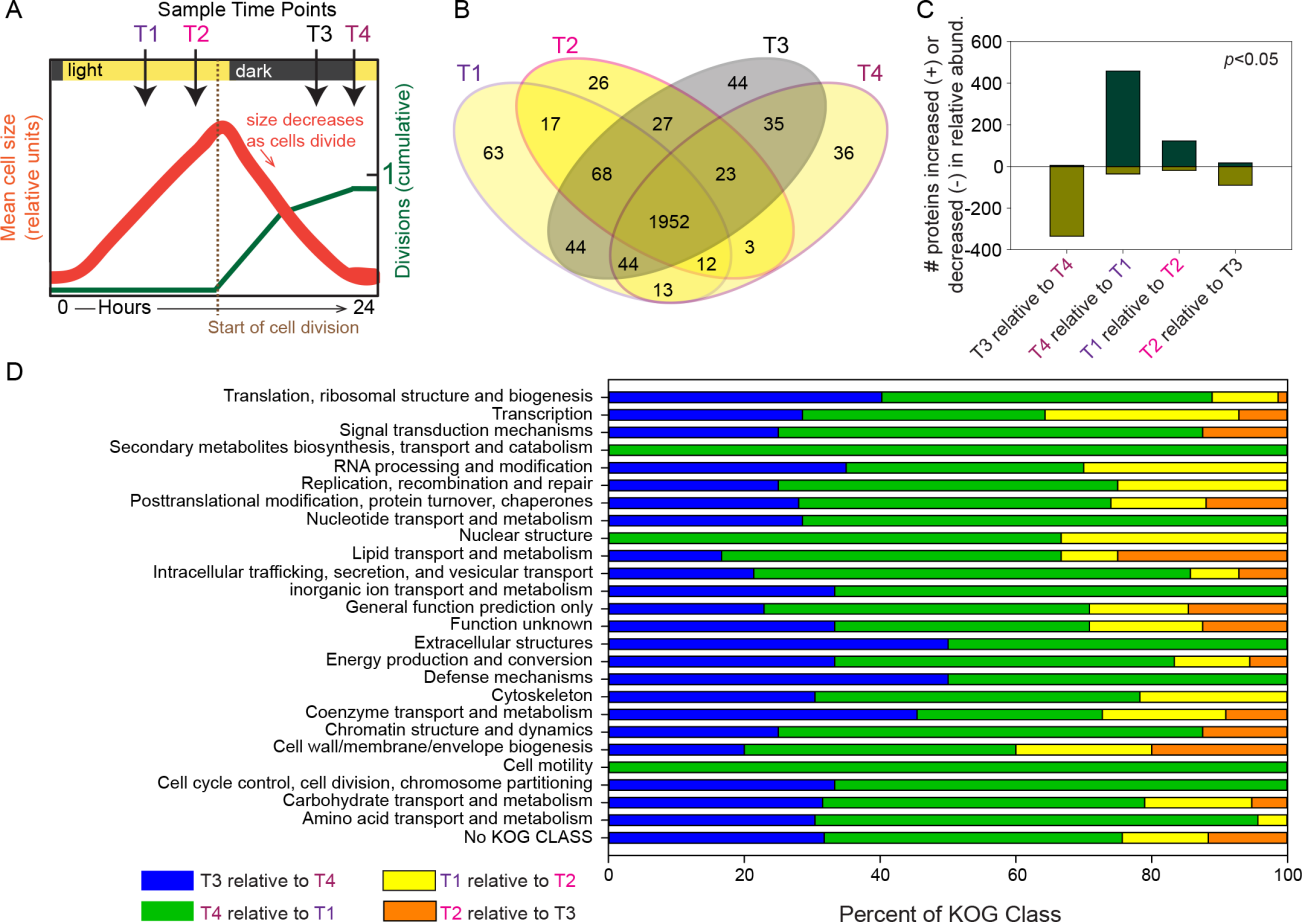
Experimental design and global dynamics of the *Micromonas pusilla* proteome. **(A)** Cartoon modeling cell growth and size during the diel experiment based on details of cell growth provided in [3]. Sampling points are marked with arrows. **(B)** Distribution across the sampled time points of proteins from the nuclear genome. Observation within at least 50% of proteomics datasets (technical and biological replicates), and identification by ≥2 unique peptides (i.e., matching only one protein in the genome) were required for categorization as “present” and inclusion here. A unique peptide is one that matches only one protein in the genome. A substantial number of proteins were observed across all time points. **(C)** Relative quantification comparison of global proteome expression over the course of the diel cycle. A progressive shift in the number of proteins exhibiting differential abundance (*p*<0.05) was observed when progressing from dark to light. The largest increased proteome response was observed at the introduction of light (T4), and this response decreased with time until relative abundance favored the dark condition. **(D)** KOG class assignments for proteins that exhibited relative abundance differences (*p*<0.05). For three KOG classes, 100% of proteins increased in abundance when comparing T4 to T1, suggesting that these classes are strongly influenced by circadian regulation in *M*. *pusilla*.

Mid-exponentially growing *Micromonas* cells were synchronized with 14:10 hr light:dark conditions so that the growth rate was 0.8 divisions d^-1^ and the majority of cells were in the same cell-cycle phase (Fig 1A). Cell size, as represented by bead normalized mean forward angle light scatter, increased throughout the photoperiod, then decreased progressively starting at dark as cells underwent division. RNA-Seq and LC-MS sampling from biological triplicates was performed 7 hours (T1) and 11 hours (T2) into the day period, the latter being 3 hours before lights out. Samples were subsequently taken during the dark period, 3 hours before lights on (termed ‘predawn’ or T3) and at the transition point between dark to light (lights on, T4) (Table 1, Fig 1A).

**Table 1:** Sample collection time points, RNA-Seq reads and proteomic spectra measured. Note that 14 hours after the initial lights on (8:00 a.m.) the lights turned off (i.e. turned off at 10 p.m.) for a 10 hour dark period that ended exactly at 24 hours past the initial lights on (i.e., T4).

Time point	T1	T2	T3	T4
**Time (hours after initial Lights On)**	+7	+12	+21	+24
**Time (actual)**	3 P.M.	8 P.M.	5 A.M.	8 A.M.
**Description**	Mid-day	Dusk	Pre-dawn	Lights On
**Peptides generated**	23,172	20,725	21,466	20,763
**Illumina reads (average of biological triplicates)**	42,576,807	41,719,472	44,085,129	40,259,370

### Proteome changes over the diel cycle

Broad scale proteomic changes were investigated over the diel cycle. Collectively, 27,840 unique peptides were generated from the four time points (T3, T4, T1, T2), which mapped to 4,176 of the 9,878 predicted proteins from the *M*. *pusilla* genome (Table 1). A subset (2,433 proteins) of the 4,176 proteins were observed in at least 50% of all replicates (technical and biological), and in at least one of the four time points. Two-thousand four hundred and seven of the 2,433 proteins were encoded on the nuclear genome. Eighty-one percent of the 2,407 proteins were observed in all time points. Thus, the number of “unique” proteins to any given time point was low (Fig 1B). Approximately 36% of the 2,407 proteins were assigned KOGS and relative representation of each broad KOG class was generally consistent across the four time points (Fig I and Table C in S1 File).

To understand the global behavior of the *M*. *pusilla* proteome over the diel cycle, proteins sampled at one time point were differentially compared to the adjacent time point in a progressive manner (Fig 1C). The proteome sampled at ‘Pre-dawn’ (T3) was largely under-expressed relative to the proteome at ‘Lights-on’ (T4), suggesting that protein expression is substantially influenced by sunrise or features entrained by the circadian clock. Nevertheless, five proteins at T3 had greater expressed abundances than at T4 (Table D in S1 File; S1 Table). These had functions related to cell proliferation, such as wlab.223673, annotated as a nucleolar family protein (NOL1/NOP2/sun family putative RNA methylase) that plays a possible regulatory role in transitioning between cell cycle phases and wlab.208730 (annotated as Aladin (Aladin/adracalin/aaas), which may regulate nucleolar activity [58–60]. The latter contains the conserved WD-repeat involved in macromolecular complex assembly [61], and may play a scaffolding role in nuclear pore complex (NPC) assembly during cell proliferation [62, 63]. With exposure to light at T4, all observed photosystem and chlorophyll production proteins increased in relative abundance compared to Pre-dawn T3. These proteins included Photosystem I and Photosystem II precursor subunits as well as light harvesting complexes. The increase in photosystem-related proteins was positively correlated (Spearman’s correlation 0.96, *p*<0.0001) with observed flagella-related proteins including flagellar protofilament ribbon protein (wlab.0195719), outer arm dynein (ODA) light chains (wlab.167555, 196373), and profilin (wlab.223036).

A progression of changes was also apparent when comparing adjacent time points during the day period as well as Dusk to Pre-dawn. At the near mid-day time point (T1) all flagella and many photosystem-related proteins had decreased in abundance relative to Lights-on (T4). This indicates potential circadian control and that *Micromonas* motility may be linked to the photocycle, or light availability, as reported in early studies of *Chlamydomonas* [64]. Proteome expression decreased when comparing Dusk (T2) to Pre-dawn T3, such that at T2 only 17 proteins were observed with higher relative abundances than T3 (Fig 1A & 1C). Among these, the nucleotide excision repair protein wlab.208745 (DNA repair/transcription protein met18/mms19) exhibited 2-fold higher expression suggesting transcription-based repair of light induced damage to DNA. Additional proteins within this group exhibiting a high degree of correlation between T2 and T3 (Spearman’s correlation 0.91, *p*<0.0001) suggest lipid and cell membrane restructuring occurs in preparation for cell division near dusk. This includes a scrambalase (wlab.223676) involved in translocation of phospholipid and squalene/oxidosqualene cyclase (wlab.203328) which is involved in the cyclization step of sterol synthesis. Also in this category is sphingosine phosphate lyase (SPL; wlab.149671), responsible for degrading sphingosine-1-phosphate (S1P) to phosphoethanolamine and hexadecenal, which are incorporated into glycerolipids. The latter are membrane constituents in many cell types–but also essential components of photosynthetic membranes in cyanobacteria and plants [65, 66]. Thus, SPL abundance at this stage in the diel may relate to chloroplast development or to modulation of sphingolipid metabolite S1P, which has broad roles including mediation of cellular processes governing growth and differentiation [67]. Additionally, we identified a chloroplast targeted diaminopimelate epimerase (DAP, wlab.209623), a protein involved in lysine synthesis (catalyzing the step to meso-DAP), with both lysine and meso-DAP being vital constituents of cell wall peptidoglycan in bacteria [68]. The role here is likely associated with chloroplast wall structure or division as *M*. *pusilla*. This species, along with several vascular plants has recently been found to encode the complete peptidoglycan biosynthesis pathway, with chloroplast targeting, although *Micromonas commoda* (represented by strain RCC299), its relatives *Ostreococcus* and *Bathycoccus*, and land plants such as *Arabidopsis thaliana*, do not [69].

### Examining the relationship between mRNA and protein expression programs

**Fig 2:**
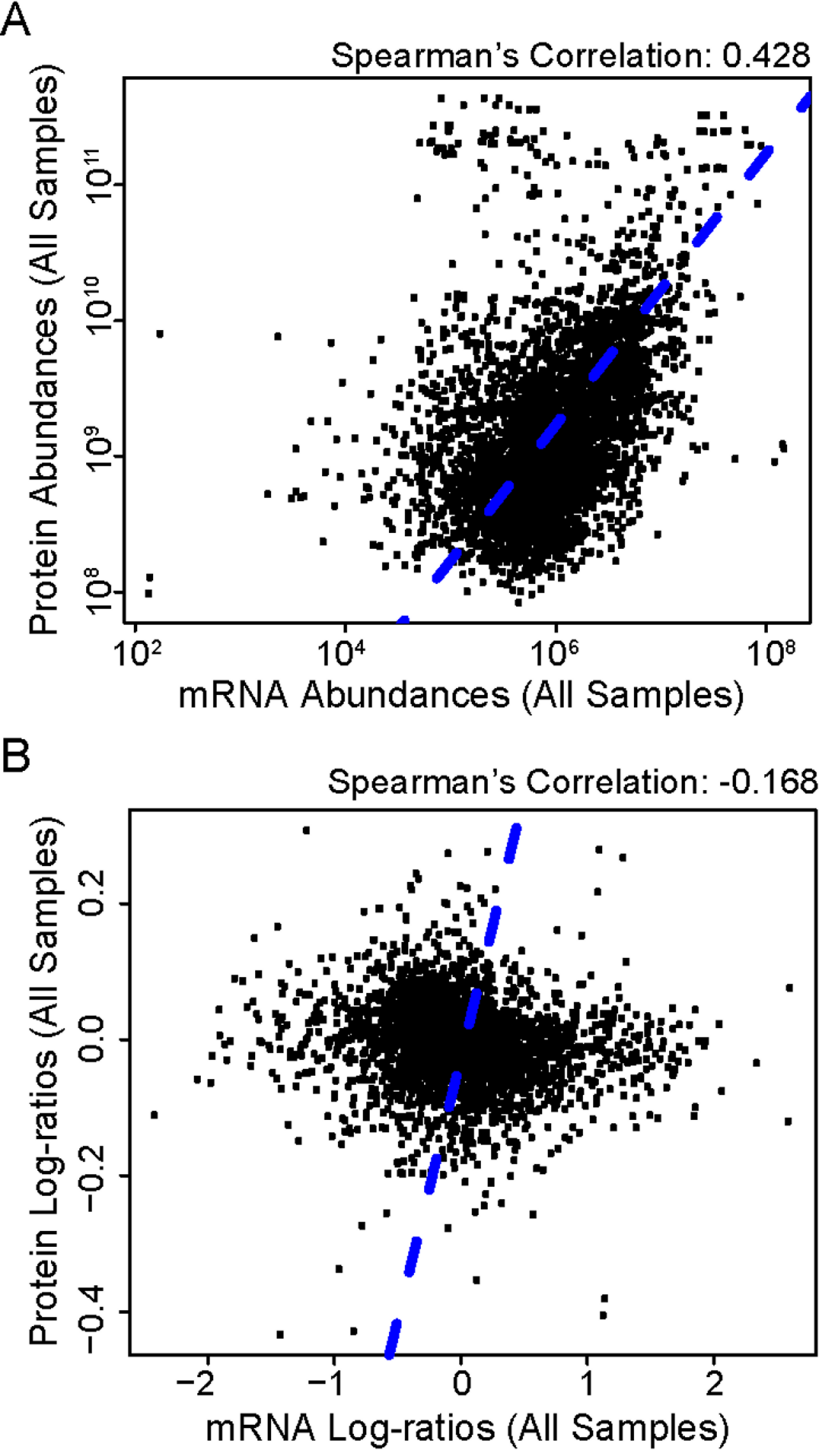
Temporal dynamics observed in mRNA and protein expression using the high-confidence set. **(A)** Comparison of absolute abundance values for the mRNA and protein expression data (all time points combined) indicates a moderate correlation between the data types (Spearman’s correlation coefficient, R_S_ = 0.428, *p*<0.0001). **(B)** Comparison of the log-ratios relative to T3 indicates a slightly negative correlation (R_S_ = -0.168, *p*<0.0001). These contrasting results suggest that while a relationship exists between mRNA and protein expression, there are considerable temporal differences between the respective expression programs.

Eleven percent of the *Micromonas* predicted proteome (1060 proteins; 25% of those detected) was represented by highly reproducible LC-MS data (Figs A-D in S1 File) and categorized as high-confidence based on quartile normalization of the LC-MS data. This high-confidence set was used for comparison to RNA-Seq data from genes encoding the same proteins (see Methods). The correlation between mRNA and protein profiles was 0.428 (Spearman’s correlation, *p*<0.0001) when all time points were pooled (Fig 2A; Fig J in S1 File for individual time points). Spearman correlations were similar (between 0.395 and 0.500, *p*<0.0001) within individual time points (Fig J in S1 File). These results are consistent with reports on *Saccharomyces cerevisiae* and *Saccharomyces pombe* [13, 20]. When log10-transformed ratio data from the light period (T1, T2 and T4) were compared to the Pre-dawn time point (T3; used as the reference) the correlation was either slightly negative (although not statistically significant) or none was observed (Fig 2B; Fig K in S1 File). Thus, while the overall absolute levels are weakly correlated, the relative changes from pre-dawn to light are not, indicating an appreciable de-coupling between regulatory programs controlling mRNA and protein levels, likely related to the anticipation of future needs related to photosynthesis. Indeed, antibody analysis of the phytochrome protein showed that its localization to the nucleus (where it interacts with transcription factors) is inversely related to the time point yielding maximum transcript levels, and levels in the total cellular protein fraction are stable over the diel cycle [3]. Thus, despite the statistically significant albeit weak correlation between the mRNA and protein abundances, the log-ratios indicate considerable temporal differences between their respective expression programs.

**Fig 3:**
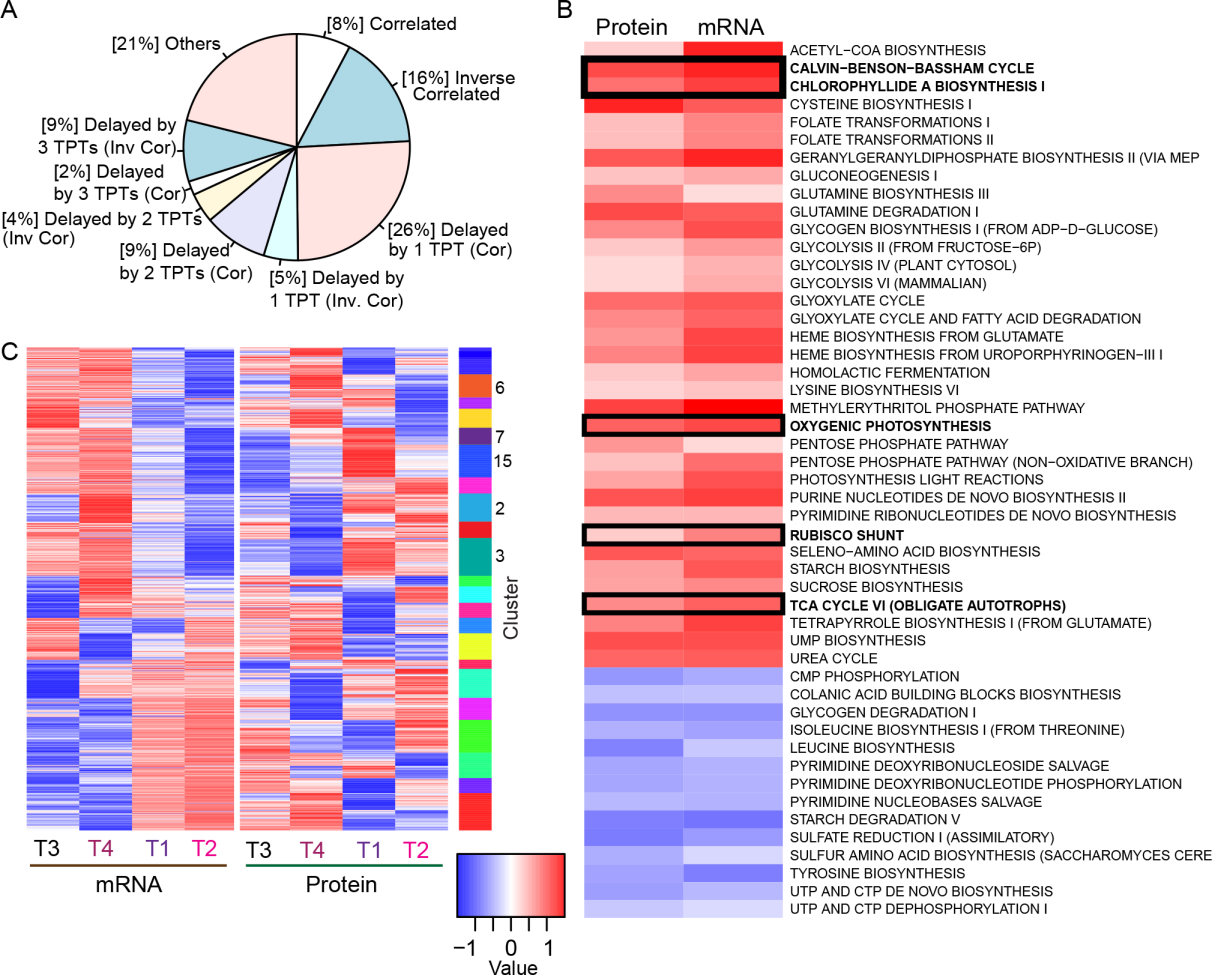
Comparison of protein and mRNA expression patterns across the time course. **(A)** Comparison of the degree of the correlation (Pearson, R_P_) between the mRNA and protein expression profiles, per gene (Z-transformed). Less than 10% of the genes considered were correlated over the course of the experiment (using a threshold of 0.75); while 26% were delayed by 1 time point (1 TPT) and 9% by 2 time points (2 TPTs). **(B)** Concordance of Gene Set Enrichment Analysis (GSEA) of pairwise correlation (as measured by *CS*_*p*_; see Methods) indicates there is considerable concordance between the expression programs of several key metabolic pathways, such as the Oxygenic Photosynthesis and TCA pathways. Note this is limited to those pathways that are concordant. Concordant pathways from a similar analysis of log-ratios include many of the same critical pathways (Fig M in S1 File). Complete representations of all pathways from the analysis of abundances and log-ratios are also provided (Figs N and O in S1 File). **(C)** A global comparison of the expression dynamics observed in the mRNA and protein expression programs.

Relationships between the mRNA and protein expression programs were examined using a gene-centric approach to identify classes of associations between mRNA and protein expression profiles over the diel. In concordance with the lack of correlation observed when log-ratio differentials were compared, only 8% (n = 82, *p*<0.0001) of the high-confidence gene set had highly correlated mRNA and protein expression profiles (Fig 3A; see material and methods for definitions) and less than 35% of the high-confidence genes (n = 361, *p*<0.0001) exhibited a slight positive correlation (Fig L in S1 File). Anti-correlated mRNA and protein expression profiles were observed for 16% of the high-confidence set (n = 174, *p*<0.0001), more than double percentage observed for highly-correlated genes. Over half had expression programs that appeared delayed by one or more time points, with over a quarter of the genes (n = 272; 25.6%; *p*< 0.0001) having protein expression profiles that were correlated, but delayed by a single time point. Another 10% were delayed by 2 or more time points (delayed by 2 time points: n = 97; delayed by 3 time points: n = 21; *p<*0.0001 for both). Nearly 20% of the genes had protein expression profiles that were delayed, but anti-correlated (*p*<0.005). These observations are consistent with Waldbauer et al [22] who sampled every 2 hours and observed that nearly 50% of the observed genes had a peak protein expression that lagged behind the peak mRNA expression by 2 to 8 hours. Similarly, Schwanhauser et al. [11], working with murine fibroblasts, demonstrated that mRNA and protein stability are highly related to function, and identified 4 classes of genes that shared similar mRNA and protein half-lives. Of these, they demonstrated that transcription factors and cyclins tend to have unstable mRNA and protein products, while products for genes and proteins of other functions are more stable. The delay observed here between mRNA and protein abundance could reflect an underlying mechanistic connection between transcriptional and translational processes. However, such a connection is likely indirect since causal relationships are thought to occur on the order of minutes [21, 70]. However, some changes observed here could also reflect cases where required protein levels influence mRNA expression, i.e. in situations where the cell increases gene transcription in anticipation of the needed protein product [71–73]. Higher resolution data might reveal different patterns that were potentially missed during the period intervening sampling time points.

### Pathway enrichment concordance between protein mRNA and protein expression

Differences in the correlation between mRNA and protein expression were tested using a gene set enrichment analysis (GSEA) [74, 75] to determine whether they reflected biological themes. GSEA measures the degree to which a particular pathway has a significant number of high-scoring proteins. Pearson correlation coefficients from the pairwise comparisons between the high-confidence genes were used as a metric and metabolic pathways predicted by PathoLogic [43, 44] were used as gene sets. To facilitate pairwise gene correlations, the pathways were converted to contain all the gene pairings possible for those in a given pathway (see Methods). We first analyzed the mRNA and protein expression data separately using GSEA, and then compared the results to evaluate concordance using a score that provided a weighted estimate of the mutual co-enrichment (or co-exclusion) of each pathway implicated by each data type. This procedure identified 35 metabolic pathways that were concordantly and positively enriched on both the mRNA and protein levels (Fig 3B; Figs M-O in S1 File). These included pathways for oxygenic photosynthesis, the Rubisco shunt, the TCA cycle, and Chlorophyllide *a* biosynthesis, indicating the presence of biologically meaningful structure in each data type detectable at the pathway level. Other photosynthetic taxa show strong patterning in connection to the diel cycle and have circadian regulation of transcription [3, 76–78]. Although fewer studies have addressed global proteomic changes, these taxa presumably have tight feedback loops controlling all protein levels, especially with respect to photosynthesis machinery.

### Identifying co-expressed gene modules

**Fig 4:**
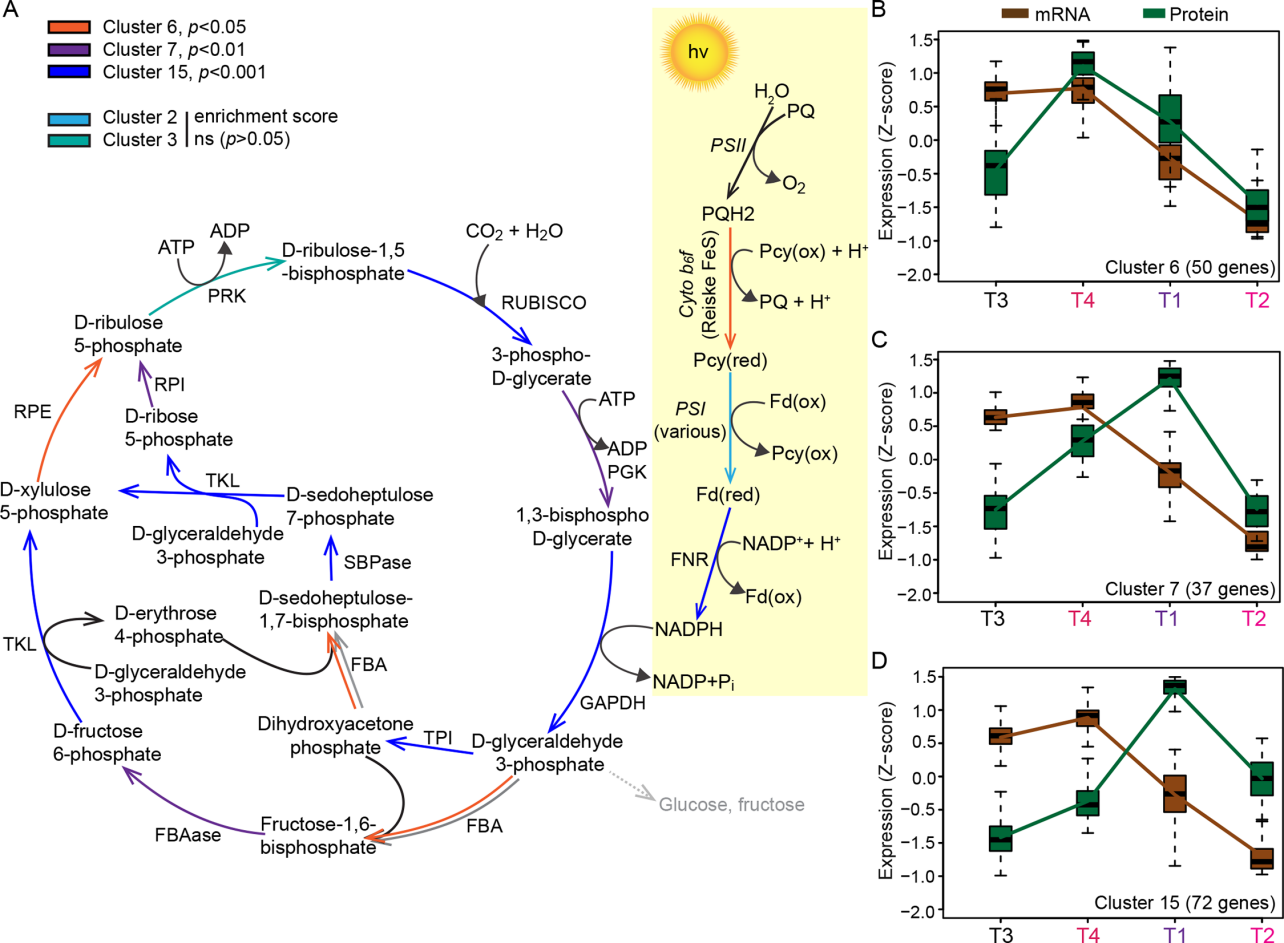
Coverage of the Oxygenic Photosynthesis (OP) Pathway by the joint expression clusters. **(A)** Cartoon of the OP pathway, with selected interactions color-coded to indicate cluster membership. Light-dependent reactions are indicated by yellow background. Of the 17 interactions in the pathway, 15 were mediated by genes in the high confidence data set (Tables E-G in S1 File). Note that within the light-independent reactions of the Calvin-Bensen-Bassham Cycle we identified two fructose-bisphosphate aldolase (FBA) proteins, wlab.223910 (Class I, Cluster 6) and wlab.149815 (Class II, Cluster 3), both with predicted chloroplast transit peptides. The Class II FBA of the cyanobacterium *Synechococcus* shows higher reactivity for sedoheptulose-1,7-bisphosphate than for fructose-1,6-bisphosphate than its Class I FBA [94] and thus, although they have not been experimentally characterized, the *M*. *pusilla* FBAs depicted here may also partition within the pathway. **(B)** Joint mRNA and protein expression profiles of the clusters enriched with OP pathway genes (Clusters 6, 7, & 15). Cluster 6 displays considerable correlation (R^2^ = 0.643) between the mRNA and protein expression patterns, while Cluster 7 and 15 display either marginal (R = 0.161) or inverse (-0.446) correlation. Profiles for Clusters 2 and 3 are show in Figure P in S1 File.

The high degree of pathway co-expression indicated by the GSEA analysis was supported by subsequent cluster analysis that identified twenty-two gene cluster modules (Fig 3C). Of the 22 clusters generated from the joint mRNA and protein expression matrix, 11 had average within-cluster, pairwise correlations of at least 0.75. Three of these, Clusters 6 (*p*<0.05), 7 (*p*<0.01) and 15 (*p*<0.001) were enriched for genes in the oxygenic photosynthesis (OP) pathway, which includes the Calvin-Benson-Bassham Cycle (CBBC) pathway responsible for catalyzing the light independent reactions of photosynthesis (converting CO_2_ to glucose) (Fig 4A). Two other clusters (2 and 3) contained multiple members of this pathway but their scores for enrichment were not significant (Fig 4A). In aggregate, the genes of these five clusters covered 15 of the 17 reaction steps in the OP pathway. Cluster 7 was also enriched for genes in sucrose biosynthesis, while clusters 6 and 15 were enriched with Chlorophyllide *a* biosynthesis I pathway genes, as was Cluster 3 (Tables E and F in S1 File and S2 Table).

Enrichments of Gene Ontology (GO) terms were also similar. Clusters 2, 3 and 15 were enriched with multiple terms involved in photosynthesis or related processes (Table G in S1 File). Of particular note, although Cluster 2 lacked statistically significant enrichment scores in the above analysis, it was enriched for the two cellular component GO terms (photosystem I and oxygen evolving complex) that are involved in those pathways. Almost all the genes in Clusters 2, 3, 6, 7 and 15 share similar mRNA expression profiles (Fig P in S1 File). Thus, the addition of the protein data drove the placement into distinct groups, suggesting a divergence in protein expression patterns. For example, for the OP pathway enriched clusters, the protein and mRNA expression profiles of Cluster 6 are highly correlated (Fig 4B), while the protein expression profiles of Clusters 7 and 15 appear to be delayed by one time point (Fig 4C and 4D). The divergent protein expression programs potentially result from differing post-transcriptional regulation, different protein turnover rates for members of these clusters, or factors such as involvement in multiple pathways or pathway steps.

### Relationships between sequence features and protein expression

To identify possible determinants of differences in temporal dynamics and connections between mRNA and protein expression programs, we constructed predictive regression models following the approach of Vogel et al. [13]. Previous studies show that gene sequence-based features provide better information for predicting protein levels than mRNA abundance alone [12, 13, 15]. Given the limited gene-wise and within sample correlations observed between the mRNA and protein abundance in our study, we hypothesized that one or more mechanisms of post-transcriptional control may be involved, while recognizing that sampling resolution may also hinder detection of relationships. The model we developed incorporated 163 sequence features as proxies for mechanisms of post-transcriptional regulation and proteomic degradation (see Methods and Table B in S1 File).

Prior to building regression models, we determined whether or not the sequence features alone had linear relationships to our data set. The Spearman correlation between a given feature and the mRNA abundance for that sample was computed for each time point. In addition, the partial correlation between the feature and protein expression was calculated, given the mRNA abundance. Correlations for the mRNA were calculated for the high-confidence set to determine if the features had any correlation with the mRNA abundances for these genes. They were also calculated for all 9,831 genes encoded in the nuclear genome to determine whether correlations observed with the high-confidence set reflected a generalized correlation to these features.

**Fig 5:**
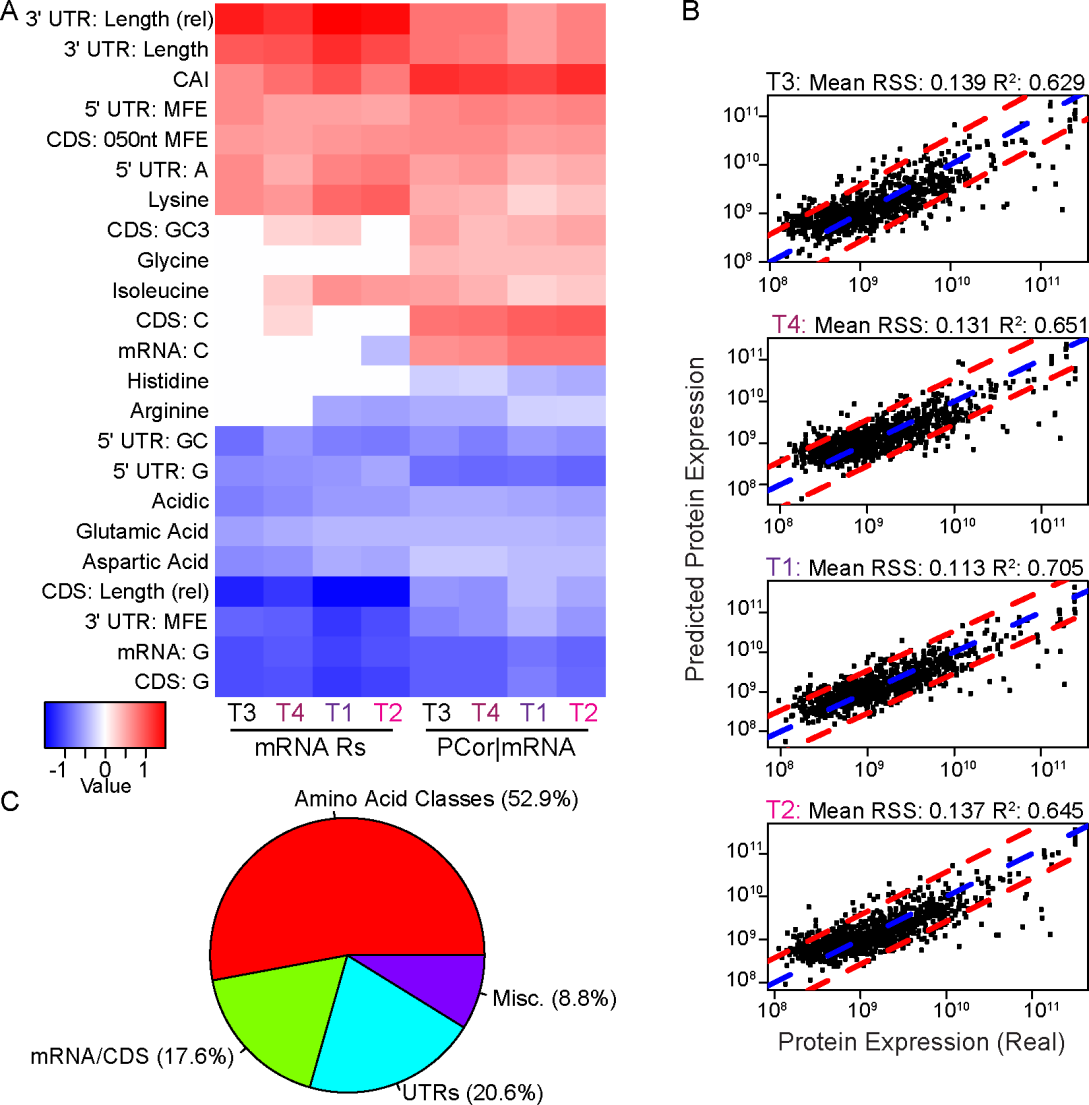
Testing the effectiveness of sequence features as proxies for post-transcriptional control. **(A)** Partial correlation matrix of the most correlated and anti-correlated features that were significantly partially correlated with protein expression in all time points, as indicated by bootstrap testing. The left four columns indicate the Spearman correlation between mRNA expression and the features per time point; the right four columns show the partial correlation of the features with the protein expression, when accounting for their correlation with the mRNA expression. As expected, features such as CDS sequence length were anti-correlated with mRNA and protein expression. An anti-correlation observed with the minimum free energy (MFE) in 3’ UTRs was notable, indicating that greater 3’ UTR structure is correlated with protein expression. **(B)** Comparison of the real versus predicted protein abundances for each sample. The blue dotted line indicates the slope and intercept for a perfect correlation (y = x); while the red dotted lines indicate the 5% and 95% quartiles for the residuals from the predicted protein expression abundances. **(C)** Categories of sequence features used in two or more of the MARS models. While features from the CDS, mRNA and UTR sequences comprised roughly 38% of the selected features, the majority were proportions of amino acid and amino acid classes in the protein sequences.

The Codon Adaptation Index (CAI) was the most positively correlated feature with mRNA abundance, having an average correlation of 0.36 (*p*<0.0001; Fig 5A; Fig Q in S1 File). CAI is a measure of a gene’s disproportionate use of particular codons reflecting the bias at the wobble position in each codon. Bias toward preferred codons (high CAI) is often proportional to the protein expression levels [79]. Here, 3’ UTR lengths were also significantly correlated to mRNA abundance (mean Rho 0.22, *p*<0.0001, Fig 5A), an effect that has been reported in plants and other multicellular eukaryotes relating to increased translational efficiency and mRNA stability of genes with longer 3’ UTRs [80]. Protein abundance was also correlated with the minimum free energy (MFE) of both the 5’ UTR (mean Rho 0.20; *p*<0.0001), as well as the first 50 nucleotides of a gene’s coding sequence (CDS; mean Rho 0.22; *p*<0.0001). Smaller MFE estimates indicate greater sequence structure, suggesting that increased structure in either of these regions is correlated with lower protein expression. This agrees with reports on other organisms that increased structure in the 5’ UTR can retard the ribosome’s progress, leading to reduced translational efficiency [13, 81]. Beyond these features, the proportions of isoleucine, glycine and lysine in the final peptide chain, as well as the proportion of cytosine in the CDS (CDS-C), the proportion of adenine in the 5’ UTR (5UTR-A) and GC-enrichment of the 3^rd^ codon position (CDS GC3) were also significantly correlated with protein expression (*p*<0.0001 for each). Three of these six features (isoleucine, lysine, 5UTR-A) were also significantly correlated (*p*<0.0001) with mRNA expression in two or more samples.

For features that had negative partial correlations, the three most anti-correlated with protein expression were measures of the proportion of guanine in the CDS, the full transcript and the 5’ UTR of a gene (mean R_P_ -0.28, -0.27 and -0.27, respectively; *p*<0.0001 for all). The GC enrichment of the 5’ UTR was also among the most anti-correlated features and has been implicated in increased RNA secondary structure in yeast [20]. Together with the above results, this is suggestive of a role for secondary structure within the 5’ UTR in regulating translation in *Micromonas*. CDS and mRNA transcript lengths were also anti-correlated with protein expression, although neither was in the top 10 most anti-correlated features. Instead, the relative length of the CDS (relative to the total transcript length) was among the most anti-correlated, as was the proportion of arginine, glutamic acid, histidine, and the acidic class of amino acids in the final peptide chain. 3’ UTR MFE was in the top 10 most anti-correlated features, suggesting increased RNA secondary structure in the 3’ UTR is associated with greater protein abundance, unlike the relationship we observed for 5’ UTR MFE. It remains unclear how greater RNA stability might influence higher protein abundance since *M*. *pusilla* lacks clear homologs for known RNA interference regulatory machinery [1].

### Predictive models of protein abundance

The partial correlation analysis revealed specific sequence features that potentially have power for predicting protein abundances. Therefore, we trained estimators of protein levels to systematically uncover combinations of sequence features important for translational regulation. The relationship between mRNA and protein expression is generally considered non-linear [13]. Consequently, we used a non-linear regression approach to model protein expression. Multi-Adaptive Regression Splines (MARS) [82, 83] outperformed other non-linear methods such as boosted regression [84] and stochastic gradient boosted trees [85] and was therefore selected as our modeling approach. A distinct MARS model was generated for each sample and its resubstitution accuracy estimated by calculating an R^2^ statistic to measure the correlation between predicted and observed protein levels on the training data such that higher R^2^ values indicate greater accuracies in terms of the variance captured (Fig 5B). Note these resubstitution R^2^ values are optimistic levels of accuracy since the same observed protein levels are used both to train and to evaluate the models. The models were capable of capturing between 62.9% and 70.5% of the total variance in the protein expression data (65.7 ± 3.3 (s.d.) %). 34 of the sequence features selected for inclusion in two or more MARS models grouped into four primary categories (Fig 5C). Categories of predictive features included the proportions of 12 amino acids and six amino acid classes. Additionally, four features were associated with 3’ UTRs and three with 5’UTR sequences. Moreover, the MARS models identified the over-representation of dinucleotide TT in 3’ UTRs as being anti-correlated with protein abundance. The TT dinucleotide is part of the AU-rich element (AREs) motif (AUUUA), and may represent a proxy measure for the presence of AREs (the binding motifs for ARE binding proteins) in the 3’ UTR. While ARE-binding proteins have been shown to promote mRNA stability in both unicellular [86] and multicellular [87] eukaryotes, they are primarily known to mediate mRNA degradation [88–91]. These observations help explain the inverse correlation between 3’ UTR length and protein abundance. Six other sequence features were associated with either mRNA transcripts or CDS, including the CAI metric and several nucleotide proportions (Table H in S1 File). There were also several miscellaneous features used in more than one of the models generated, including the hypothetical isoelectric point of the genes, the proportion of coils within the peptide sequence (as predicted by DisEMBL), and chloroplast localization predictions from TargetP.

Ten-fold cross-validation was performed to determine the generalizability of the MARS models that we generated. This analysis resulted in a considerable decrease in prediction accuracy compared to the resubstitution accuracies described above (Fig R in S1 File). The levels observed were comparable to other reduced-feature models [13], with a minimum R^2^ of 0.306 (T4), and a maximum of 0.362 (T3). This reduced accuracy may stem from overall noise in the data or computationally derived sequence features. For example, 3,101 genes (including 91 in the high confidence set) had either missing or truncated 3’ UTR predictions, while another 4,042 (including 202 in the high confidence set) had missing or truncated 5’ UTR predictions. For these genes, both improved UTR predictions and improved *in silico* predictions for RNA structure could lead to improved protein abundance predictions. We also found that many of the genes that were poorly predicted for one time point (those associated with high residuals) were also poorly predicted for other time points (Fig 5B). We hypothesized that protein expression of these genes might be determined by an alternate regulatory program, such as a post-transcriptional regulatory process. Because previous work has demonstrated that mRNA and protein stability is highly variable and associated with differences in biological function [11], we also considered that these hypothetically post-transcriptionally regulated genes (HPTR) might constitute different subsets of genes. If protein and mRNA stabilities within possible different subsets are sufficiently different from those of others, this would also negatively impact the accuracy of our MARS modeling strategy.

We identified HPTR genes among the high confidence genes as those with either under-estimated protein levels (residuals lower than the 5% quantile) or over-estimated protein levels (residuals higher than the 95% quantile) in two or more time-point-specific MARS models (Fig 5B). We identified 97 such HPTR genes with protein expression levels poorly fit by the canonical models built from the high-confidence set. HPTR gene specific models failed to generalize in cross-validation, suggesting that information needed to model the protein abundance of HPTR gene products is missing from the dataset. The 97 HPTR genes represent a diverse collection from 47 different pathways, with no single pathway showing specific overrepresentation according to enrichment analysis. Using GO term enrichments, several significant enrichments were observed (Table I in S1 File). However, these applied to only 10 of the 97 HPTR genes; and 25 out of the total 97 lacked any GO annotations. For this reason, HPTRs appear to represent either multiple functional classes or proteins of unknown function.

Our results are consistent with the hypothesis that the HPTR genes are under an alternate regulatory program. The cross-validation accuracy increased on average by 23% (*p*<0.0002) when HPTR genes were excluded from consideration such that R^2^ ranged from 0.82 to 0.83 for the four time points (Fig S in S1 File). In a more rigorous test, we selected the set of genes excluded from model training (i.e., “unseen” genes, n = 236) for cross-validation due to having incomplete feature sets. Of these 236 high-confidence genes that had been excluded from model building, 79 had sufficient feature sets for testing the trained models. Again, when compared to the original models, the models generated after excluding HPTR genes were significantly better (*p*<0.0001) at predicting the abundance of unseen genes (Fig T in S1 File; R^2^ increased by 22% so that it ranged from 0.60 to 0.68 for the four time points). This improvement further supports the notion that the relationship between mRNA and protein abundance of the HPTR genes follows a different set of rules than the rest of the genome.

Rates of protein and mRNA degradation are potentially important factors for relating transcript and protein expression programs that were not accounted for here. Schwanhäusser et al. [11] demonstrated that both mRNA and protein stability were highly associated with biological function. Their analysis indicates that genes such as transcription factors and cyclins typically have both unstable mRNAs and proteins, likely due to the necessity of their gene products being quickly degraded. In contrast, genes involved in key processes, such as cell replication machinery or major metabolic processes typically had the most stable gene products (both mRNA and proteins). Thus, degradation of both mRNA and protein represent highly regulated processes that could help connect the levels of observed mRNA to those of observed protein. Experimental estimates of mRNA and protein degradation rates are not yet available for *M*. *pusilla*. Therefore, we explored the extent to which degradation could help explain the mRNA-protein connection by testing inclusion of inferred *in silico* estimates of protein stability from ProtParam [92, 93]. To this end, we used ProtParam’s estimates as proxies for protein half-lives included as additional features to MARS training. These inferred protein stability estimates did not improve the MARS models. However, the original method by which these inferences (ProtParam based) were generated was based upon a sample set of what appears to be less than 50 human and yeast proteins [93]. Thus, despite the lack of improvement observed here, degradation effects may very well play a role in linking the mRNA to protein in *Micromonas*.

## Conclusions and Future Directions

We set out to identify diel-associated regulatory programs governing transcription and translation in *Micromonas*. Our experiments made use of a synchronized population for which the majority of cells were in the same cell-cycle phase, enabling us to monitor dynamic mRNA and protein levels and search for genes enriched for post-transcriptional control. By fitting regression models based on mRNA and nucleic acid sequence, we could identify genes with predictable protein levels using a regression-based approach. Furthermore, we expect that those genes with protein levels less predictable by the regression strategy were regulated by post-transcriptional mechanisms. These HPTR genes lack key DNA-encoded and transcriptional level features that inform the models about protein expression. Our results emphasize that these genes resist straightforward categorization into known gene functional groups based on Gene Ontology and KEGG overlap analysis. Additionally, because no single expression-based cluster is overrepresented among the HPTR genes, it appears that their expression patterns cover a diverse set of dynamics. Given that the high-confidence gene set covers only 11% of the entire predicted proteome, it is possible that patterns would emerge if the data set had more complete genomic coverage.

We have shown that signals in the mRNA sequence, in addition to mRNA abundance, confer information in *cis* to post-transcriptionally regulate protein expression for a large proportion of genes. Experimentally verified rates of transcription, translation and degradation are lacking for many organisms, including *M*. *pusilla*. Inclusion of experimental estimates of these rates would likely increase the accuracy of the model predictions. It is therefore possible that the list of HPTR genes is somewhat enriched for complex post-transcriptional regulatory mechanistic logic unexplained by *cis* effects captured by sequence, mRNA or degradation estimates. Our analysis sheds light on the complex patterns between gene and protein expression that exist in environmentally relevant species. We have now revealed several mechanisms of post-transcriptional control in *Micromonas* that have been reported for other eukaryotes. These include ribosomal efficiency, nucleotide availability, amino acid availability and 3’ UTR length. We also observed an influence of 3’ UTR structure, which to our knowledge has not been previously documented. Further studies of these cellular regulatory processes will deepen our understanding of how algae may respond to changes in the marine environment and potentially aid understanding of these processes in the plant lineage as a whole.

## Supporting Information

S1 File Supplementary Information. This file contains supplementary methods, as well as supplementary figures A-U, and tables A-J.
(DOCX)

S1 Table Progressive differential protein expression. This table contains a detailed list of differentially expressed genes, as determined by a progressive scan where protein expression from one time point was compared with data from the subsequent time point just prior to it, e.g. T2 compared with T3. In the case of T4, this was compared with the data from T1.
(XLSX)

S2 Table Oxygenic Photosynthesis clusters. This table contains information regarding the clusters that were either enriched or contained genes from the Oxygenic Photosynthesis (OP) pathway, including gene name, as well as GO term and EC number annotations. Note, EC numbers were annotated using Pathway Tools, as described in the Materials and Methods section.
(XLSX)
