# Supplementary material for: Identifying Aspects of the Post-Transcriptional Program Governing the Proteome of the Green Alga Micromonas pusilla
Source: PLoS One. 2016 Jul 19;11(7):e0155839. doi: 10.1371/journal.pone.0155839 (PMC4951065; doi:10.1371/journal.pone.0155839)
Supplement: S1 File — This file contains supplementary methods, as well as supplementary figures A-U, and tables A-J. (DOCX) [file pone.0155839.s001.docx]

**Waltman et al. Supplementary Figures, Tables, Materials & Methods and Citations**

Table of Contents

[Supplementary Figures 3](#_Toc451535920)

[Figure A: Heatmap of Pearson correlation for peptide intensities measured from sample T3.. 3](#_Toc451535921)

[Figure B: Heatmap of Pearson correlation for peptide intensities measured from sample T4. 4](#_Toc451535922)

[Figure C: Heatmap of Pearson correlation for peptide intensities measured from sample T1 5](#_Toc451535923)

[Figure D: Heatmap of Pearson correlation for peptide intensities measured from sample T2.. 6](#_Toc451535924)

[Figure E: GLM model results when using mRNA and CAI as only predictive features. 7](#_Toc451535925)

[Figure F: MARS model results when only using mRNA and CAI as predictive features. 8](#_Toc451535926)

[Figure G: GLM model results when using mRNA as sole predictive feature. 9](#_Toc451535927)

[Figure H: MARS model results when using mRNA as sole predictive feature 10](#_Toc451535928)

[Figure I: Comparison of GO term representation across the time points. 11](#_Toc451535929)

[Figure J: Distribution of mRNA and protein absolute expression (abundance), per sample. 12](#_Toc451535930)

[Figure K: Distribution of mRNA and protein relative expression (relative to T3), per sample. 13](#_Toc451535931)

[Figure L: Inverse CDF of the distribution of Pearson Correlation coefficients. 14](#_Toc451535932)

[Figure M: Pathway Concordance GSEA signatures for Log-ratios for concordant pathways. 15](#_Toc451535933)

[Figure N: Pathway Concordance between GSEA signatures for Abundances for all pathways 15](#_Toc451535934)

[Figure O: Pathway Concordance between GSEA signatures for Log-Ratios for all pathways 16](#_Toc451535935)

[Figure P: Expression profiles for the Oxygenic Photosynthesis (OP) pathway. 17](#_Toc451535936)

[Figure Q: Partial Correlation Heatmap. 18](#_Toc451535937)

[Figure R: Gene-wise cross-validation MARS model prediction results. 19](#_Toc451535938)

[Figure S: MARS models when genes in the HPTR set are excluded. 20](#_Toc451535939)

[Figure T: Cross-validated (10-fold) MARS models when genes in the HPTR set are excluded. 21](#_Toc451535940)

[Figure U: Distribution of Balanced Success Rates for the binary classifiers generated for the clusters identified. 22](#_Toc451535941)

[Supplementary Tables 23](#_Toc451535942)

[Table A: Summary of pathways inferred from PathoLogic for *Micromonas pusilla*. 23](#_Toc451535943)

[Table B: Sequence features used as proxies for mechanisms of post-transcriptional control 23](#_Toc451535944)

[Table C: Total number of differentially expressed proteins, relative to previous time point, per KOG class 24](#_Toc451535945)

[Table D: Number of differentially expressed proteins, relative to previous time point. 24](#_Toc451535946)

[Table E: Listing of enriched metabolic pathways for clusters 3, 6, 7 and 15. 25](#_Toc451535947)

[Table F: Gene lists for the clusters that contain genes in the Oxygenic Photosynthesis (OP) pathway. 25](#_Toc451535948)

[Table G: Listing of GO term enrichments for clusters enriched with Oxygenic Photosynthesis clusters (2, 3, 7, 15). 27](#_Toc451535949)

[Table H: List of MARS sequence features used (50+% of samples). 27](#_Toc451535950)

[Table I: GO Enrichments of the HPTR genes 28](#_Toc451535951)

[Table J: Features that are significantly different for the Oxygenic Photosynthesis clusters (clusters 6, 7 and 15). 28](#_Toc451535952)

[Supplementary materials and methods 29](#_Toc451535953)

[Preparation of Proteomics Data 29](#_Toc451535954)

[Peptide Analysis by Liquid Chromatography Tandem Mass Spectrometry (LC-MS/MS). 29](#_Toc451535955)

[Peptide sequence identification 29](#_Toc451535956)

[Expression-based cluster classification 29](#_Toc451535957)

Citations…………………………………………………………………………………………………………………………………………………….31

# Supplementary Figures

### **Figure A:** Heatmap of Pearson correlation for peptide intensities measured from sample T3. Heatmap includes biological replicates (BR1-BR3) and technical replicates (TR1-TR4). The lowest Pearson correlation value observed between any two datasets of peptides was 0.8, observed with T1 (Fig. C).


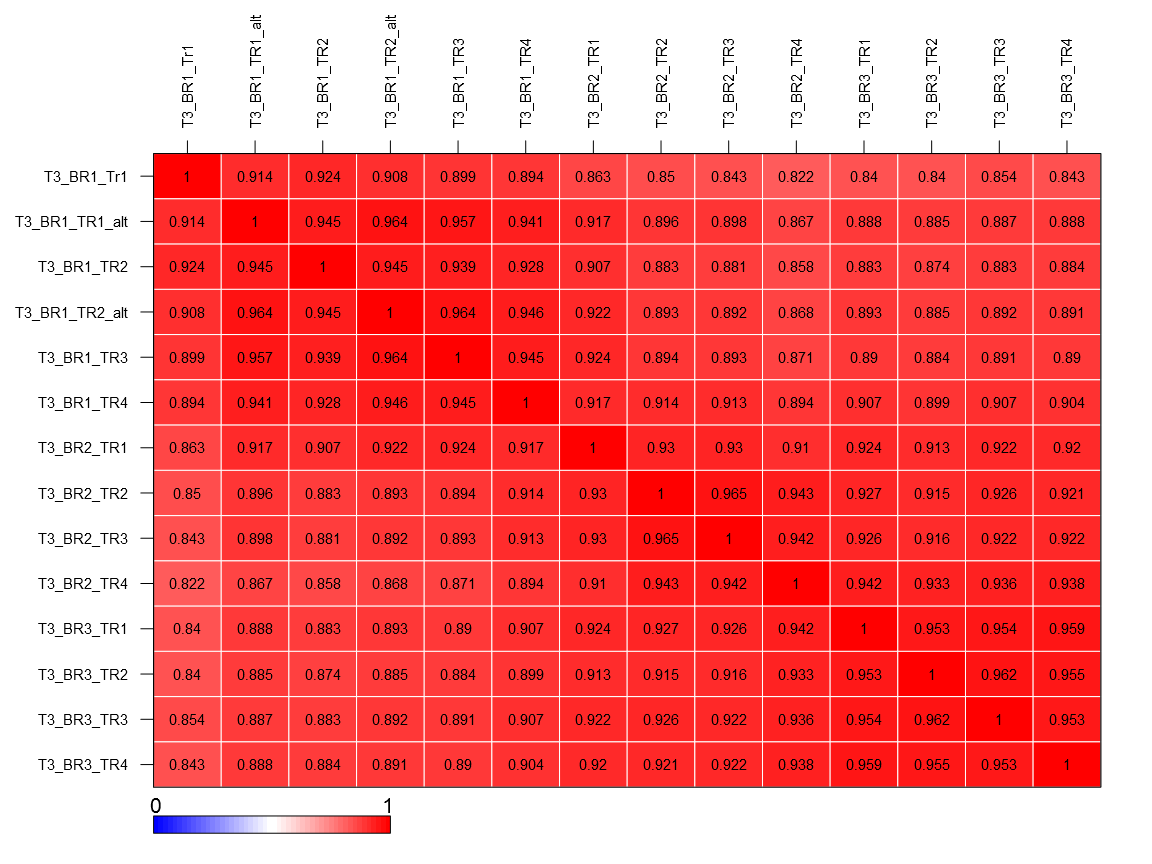


### **Figure B:** Heatmap of Pearson correlation for peptide intensities measured from sample T4. Heatmap includes biological replicates (BR1-BR3) and technical replicates (TR1-TR4). The lowest Pearson correlation value observed between any two datasets of peptides was 0.8, observed with T1 (Fig. C).


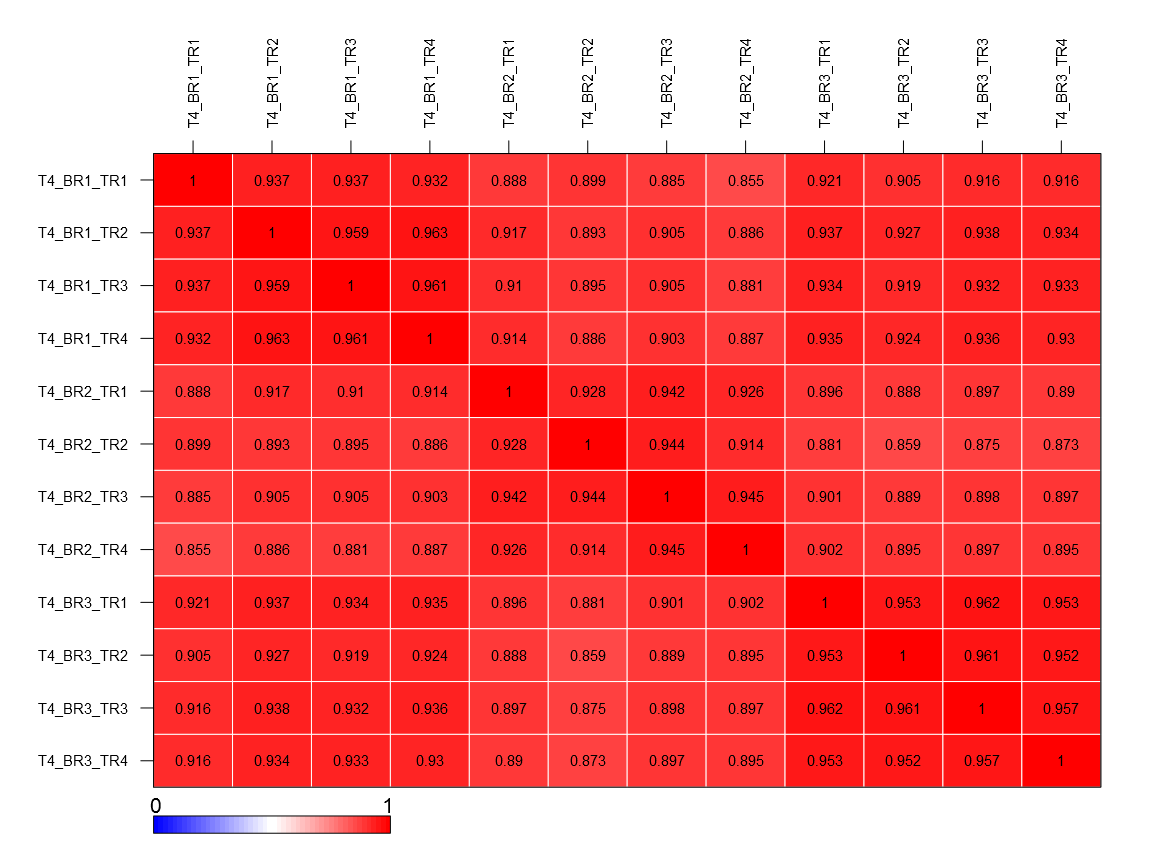


### **Figure C:** Heatmap of Pearson correlation for peptide intensities measured from sample T1. Heatmap includes biological replicates (BR1-BR3) and technical replicates (TR1-TR4). The lowest Pearson correlation value observed between any two datasets of peptides was 0.8, observed in this sample.


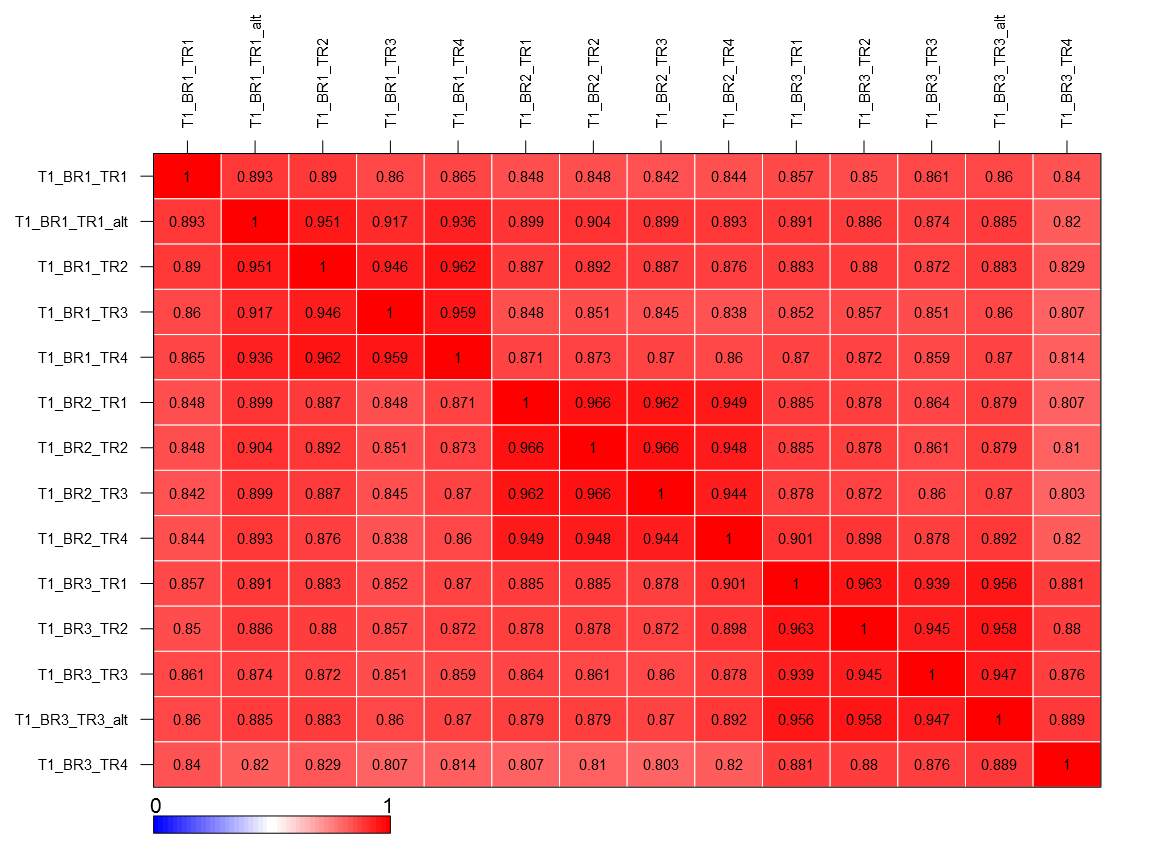


### **Figure D:** Heatmap of Pearson correlation for peptide intensities measured from sample T2. Heatmap includes biological replicates (BR1-BR3) and technical replicates (TR1-TR4). The lowest Pearson correlation value observed between any two datasets of peptides was 0.8, observed with T1 (Fig. C).


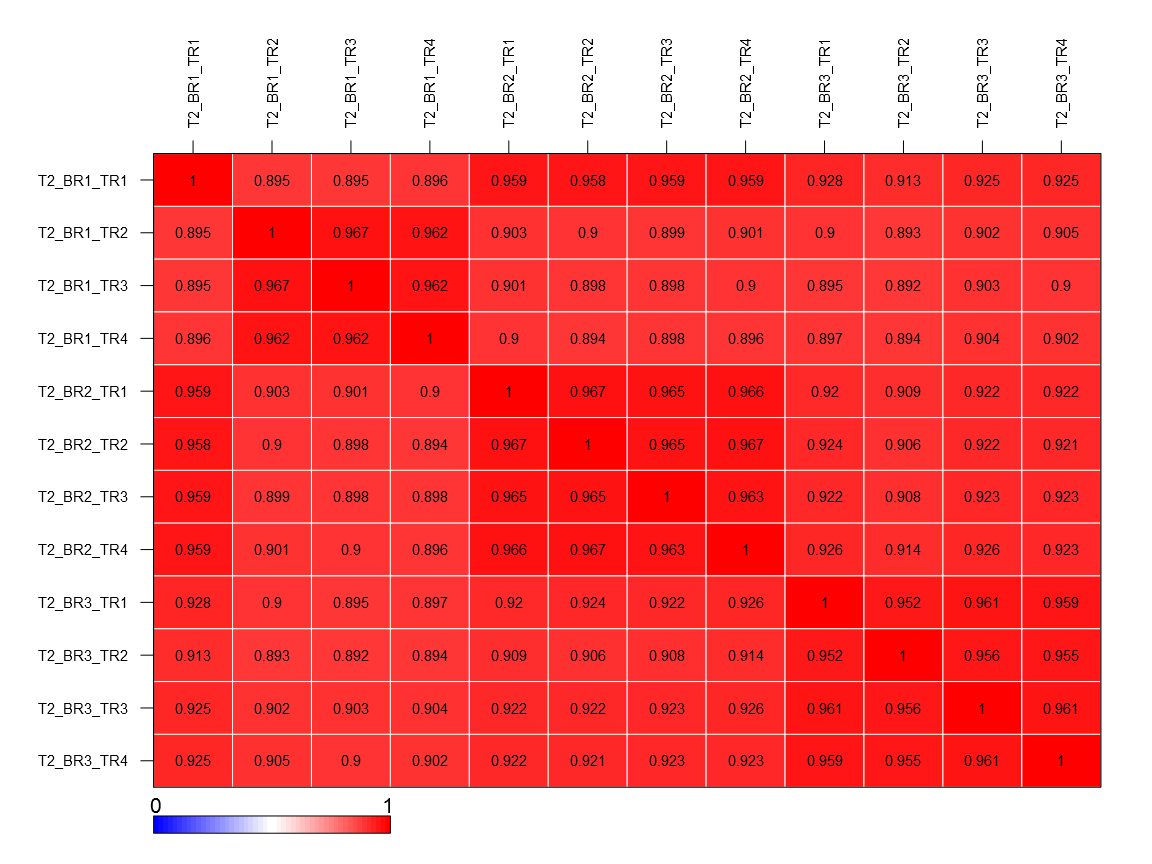


### **Figure E: GLM model results when using mRNA and CAI as only predictive features**.


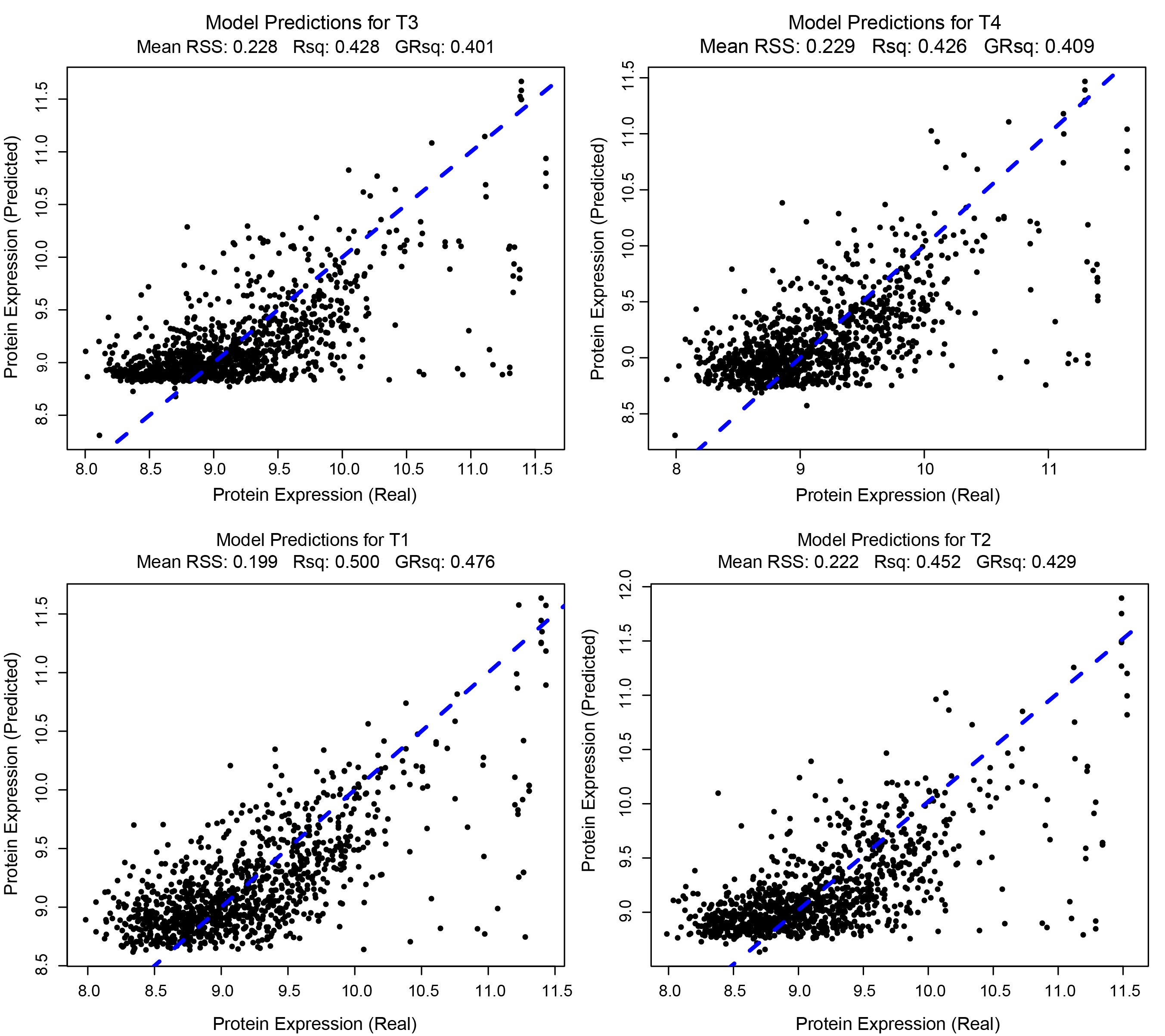


### **Figure F: MARS model results when only using mRNA and CAI as predictive features.**


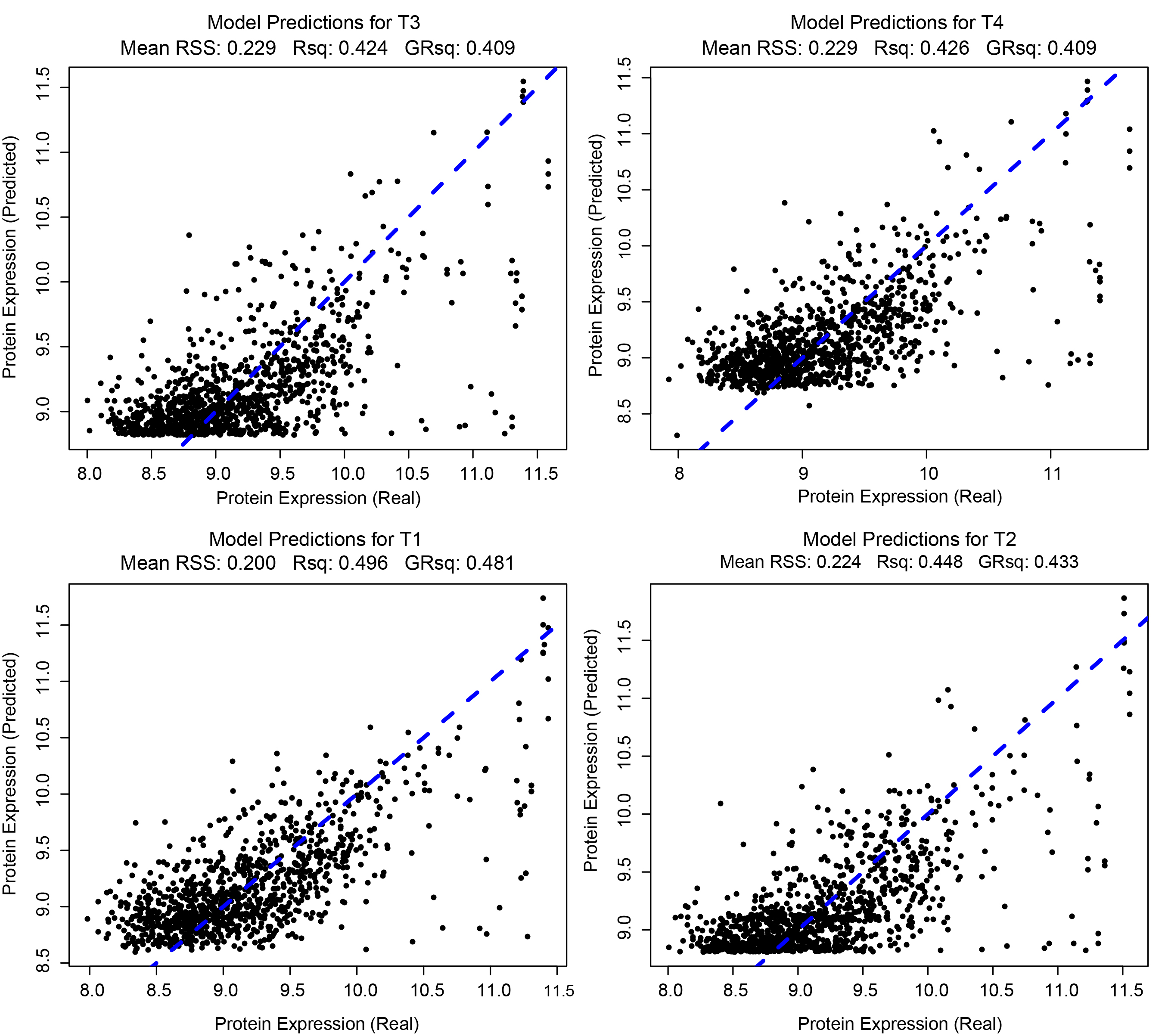


### **Figure G: GLM model results when using mRNA as sole predictive feature.**


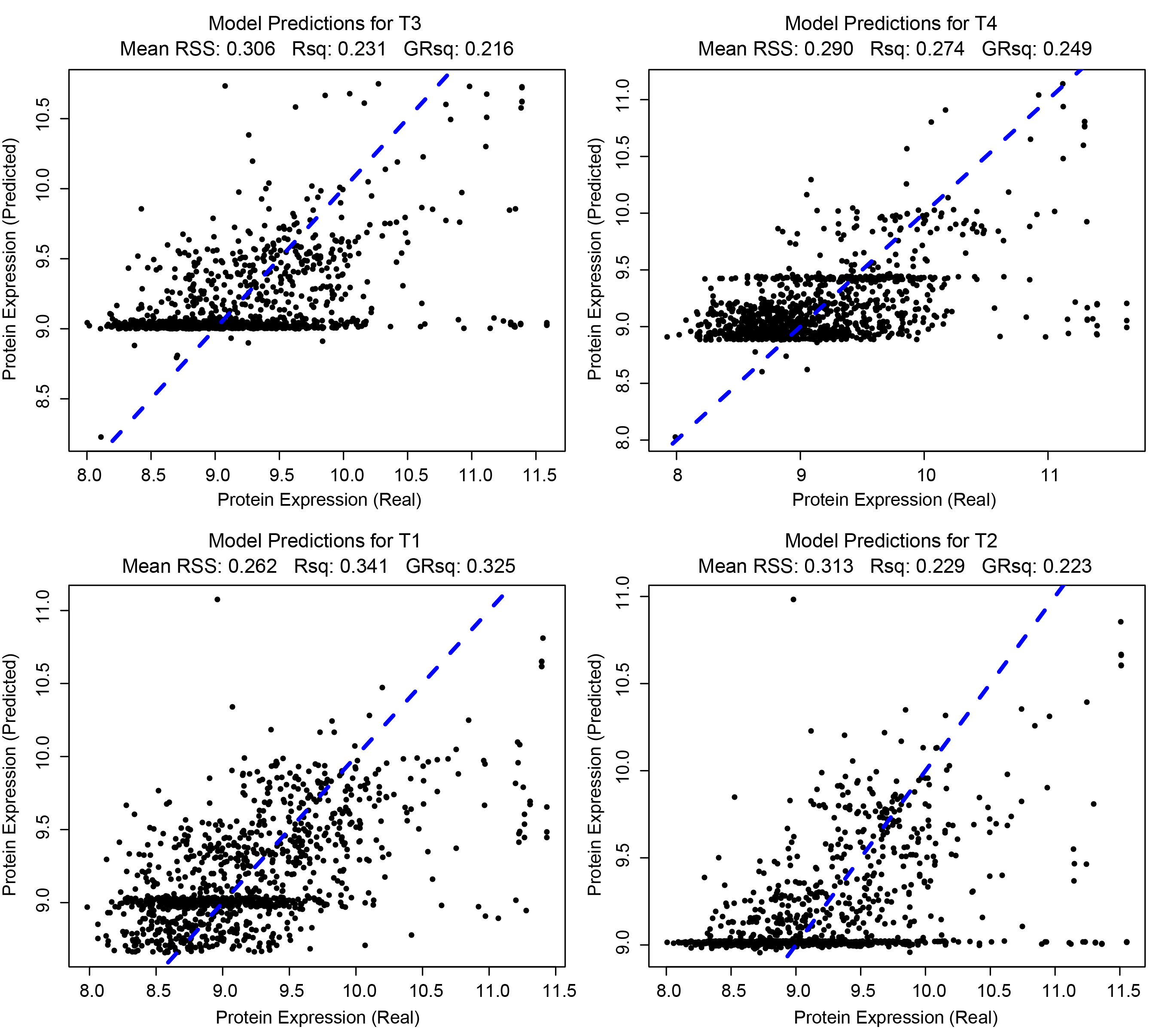


### Figure H: MARS model results when using mRNA as sole predictive feature


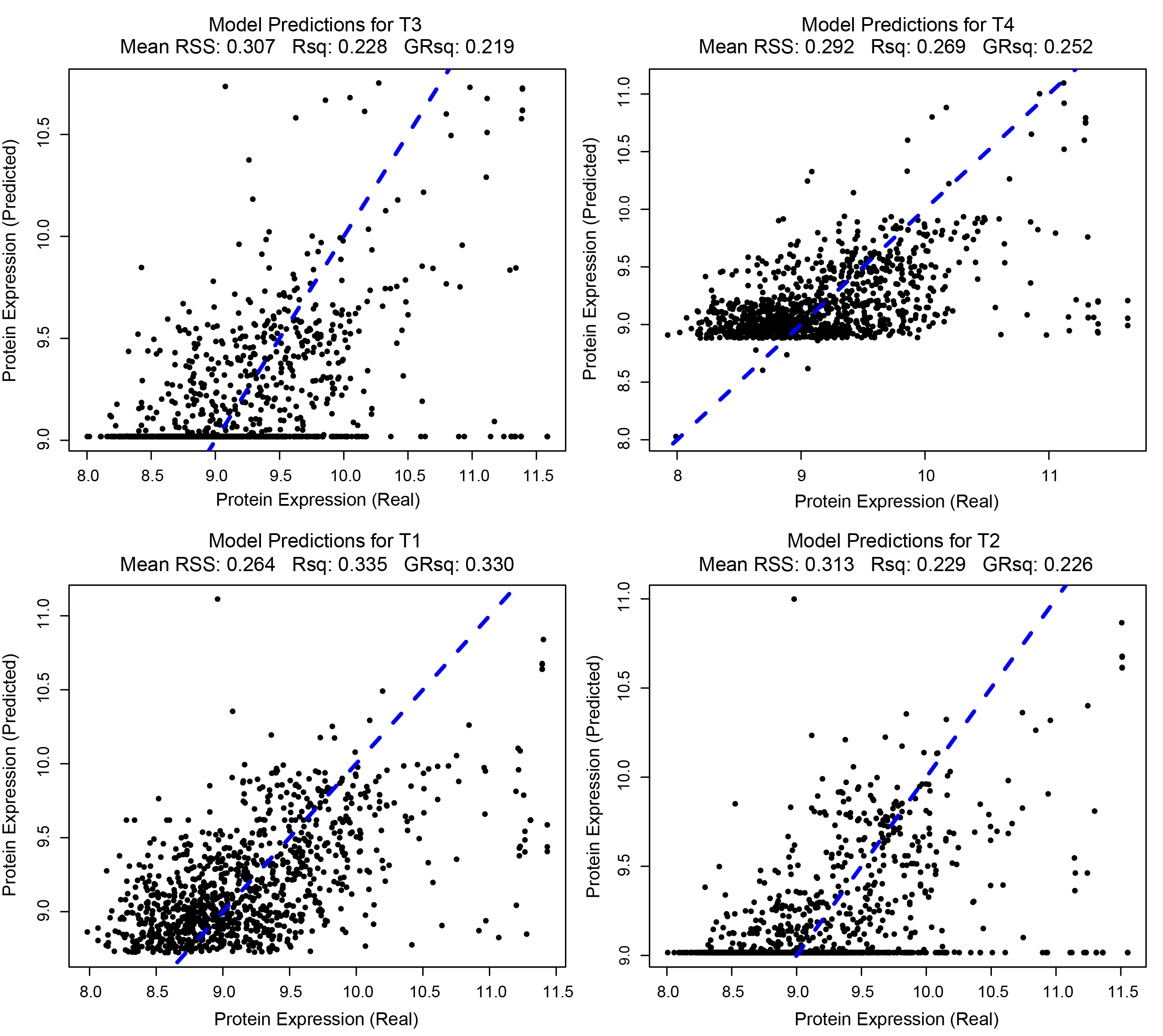


### Figure I: Comparison of GO term representation across the time points.


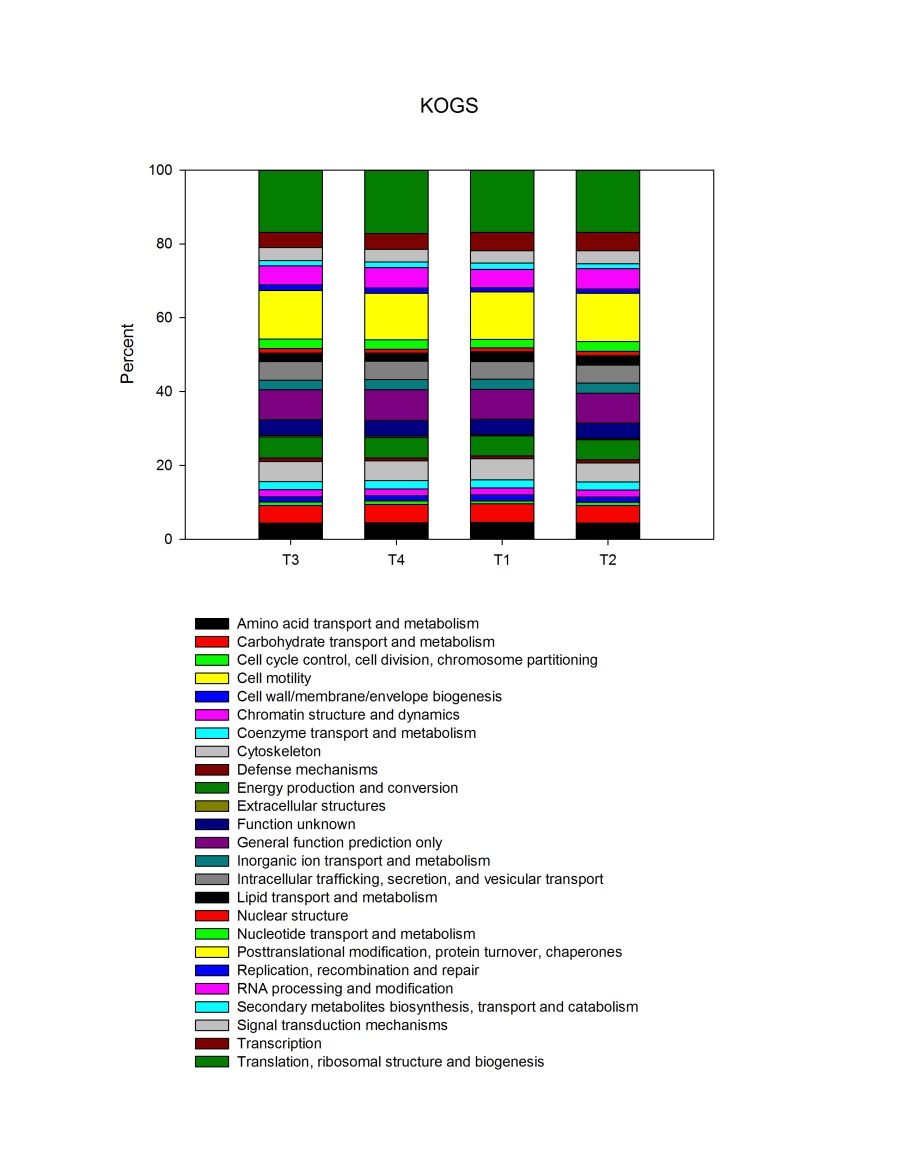

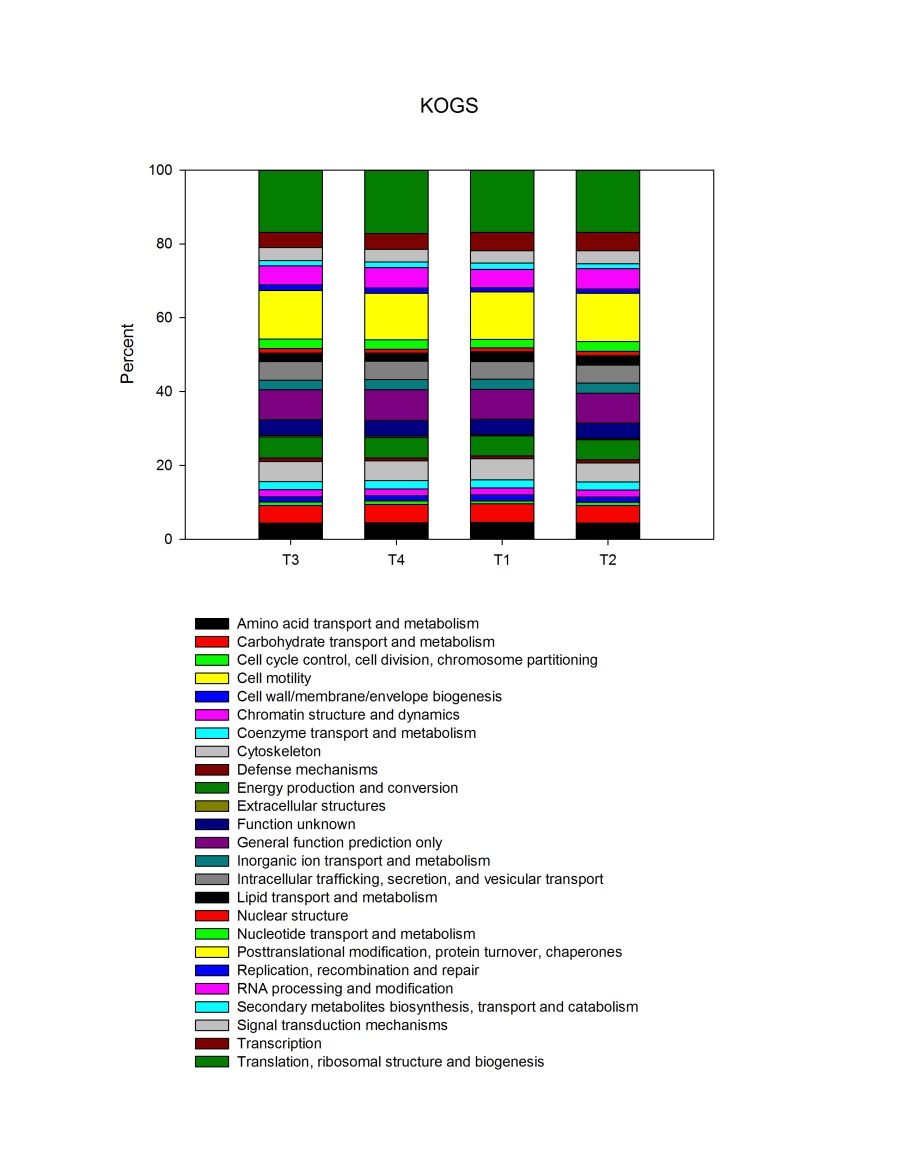


**Data used to construct Fig. I (Percent of proteins observed within a given KOG class for each time point).**

| **KOG Class** | **T3** | **T4** | **T1** | **T2** |
| --- | --- | --- | --- | --- |
| Amino acid transport and metabolism | 4.5 | 4.6 | 4.5 | 4.5 |
| Carbohydrate transport and metabolism | 5.1 | 5.0 | 5.4 | 5.0 |
| Cell cycle control, cell division, chromosome partitioning | 0.9 | 0.8 | 0.8 | 0.9 |
| Cell motility | 0.1 | 0.0 | 0.1 | 0.0 |
| Cell wall/membrane/envelope biogenesis | 1.4 | 1.4 | 1.5 | 1.4 |
| Chromatin structure and dynamics | 2.0 | 2.0 | 1.9 | 1.9 |
| Coenzyme transport and metabolism | 2.2 | 2.2 | 2.3 | 2.3 |
| Cytoskeleton | 5.1 | 5.5 | 5.4 | 5.4 |
| Defense mechanisms | 0.8 | 0.8 | 0.7 | 0.8 |
| Energy production and conversion | 5.8 | 5.9 | 5.6 | 5.6 |
| Extracellular structures | 0.3 | 0.3 | 0.3 | 0.3 |
| Function unknown | 4.2 | 4.3 | 4.3 | 3.9 |
| General function prediction only | 7.9 | 7.4 | 7.4 | 7.9 |
| Inorganic ion transport and metabolism | 2.6 | 2.8 | 2.8 | 2.7 |
| Intracellular trafficking, secretion, and vesicular transport | 5.1 | 5.2 | 5.2 | 5.0 |
| Lipid transport and metabolism | 2.3 | 2.2 | 2.5 | 2.7 |
| Nuclear structure | 1.4 | 1.0 | 1.0 | 0.9 |
| Nucleotide transport and metabolism | 2.6 | 2.4 | 2.3 | 2.3 |
| Posttranslational modification, protein turnover, chaperones | 13.1 | 13.2 | 12.8 | 13.6 |
| Replication, recombination and repair | 1.4 | 1.3 | 0.7 | 1.2 |
| RNA processing and modification | 4.6 | 4.8 | 4.7 | 5.3 |
| Secondary metabolites biosynthesis, transport and catabolism | 1.2 | 1.4 | 1.8 | 1.5 |
| Signal transduction mechanisms | 3.4 | 3.4 | 3.2 | 3.1 |
| Transcription | 4.5 | 4.1 | 4.7 | 4.5 |
| Translation, ribosomal structure and biogenesis | 17.5 | 18.1 | 18.0 | 17.3 |

### Figure J: Distribution of mRNA and protein absolute expression (abundance), per sample.


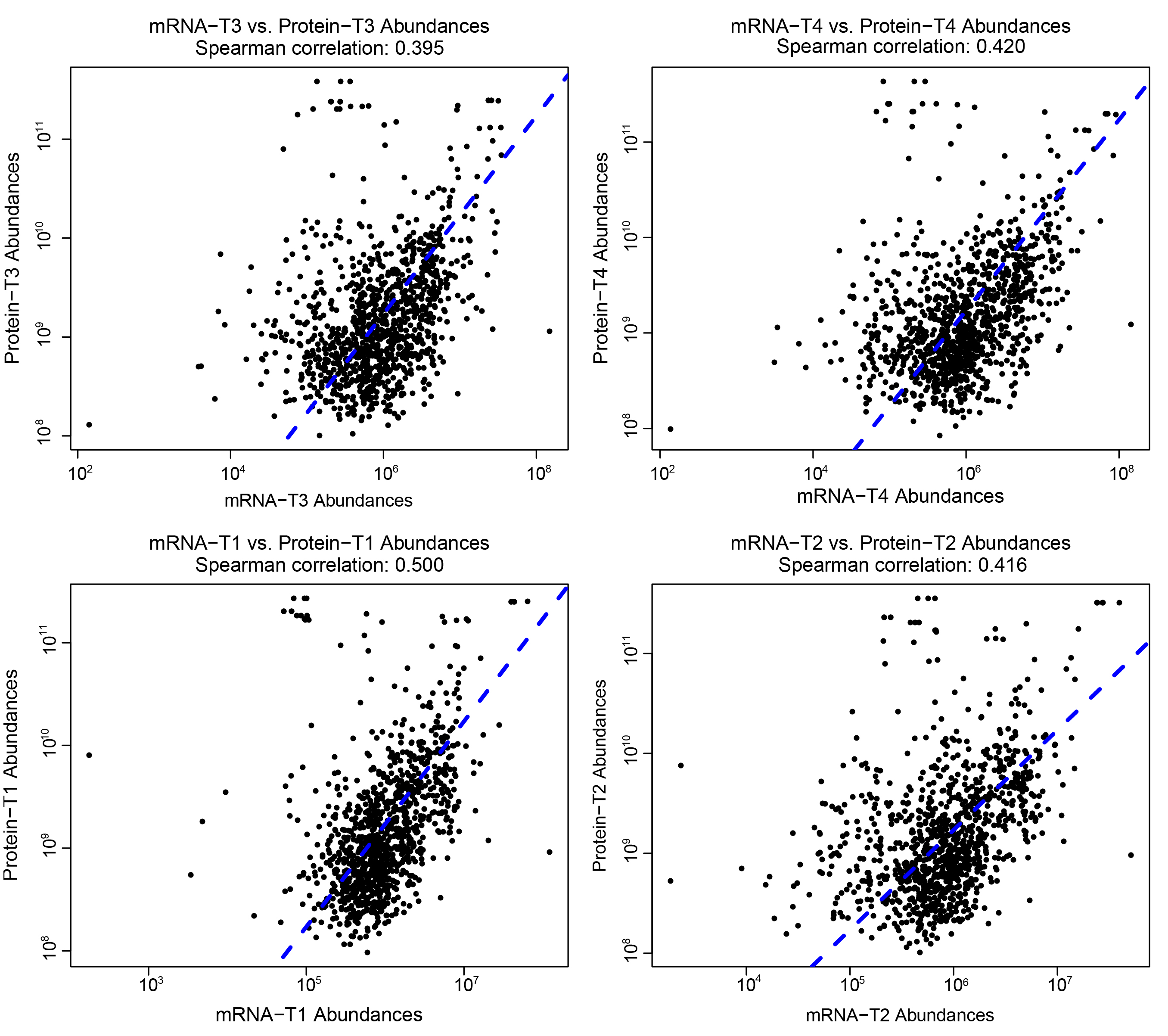


### Figure K: Distribution of mRNA and protein relative expression (relative to T3), per sample.


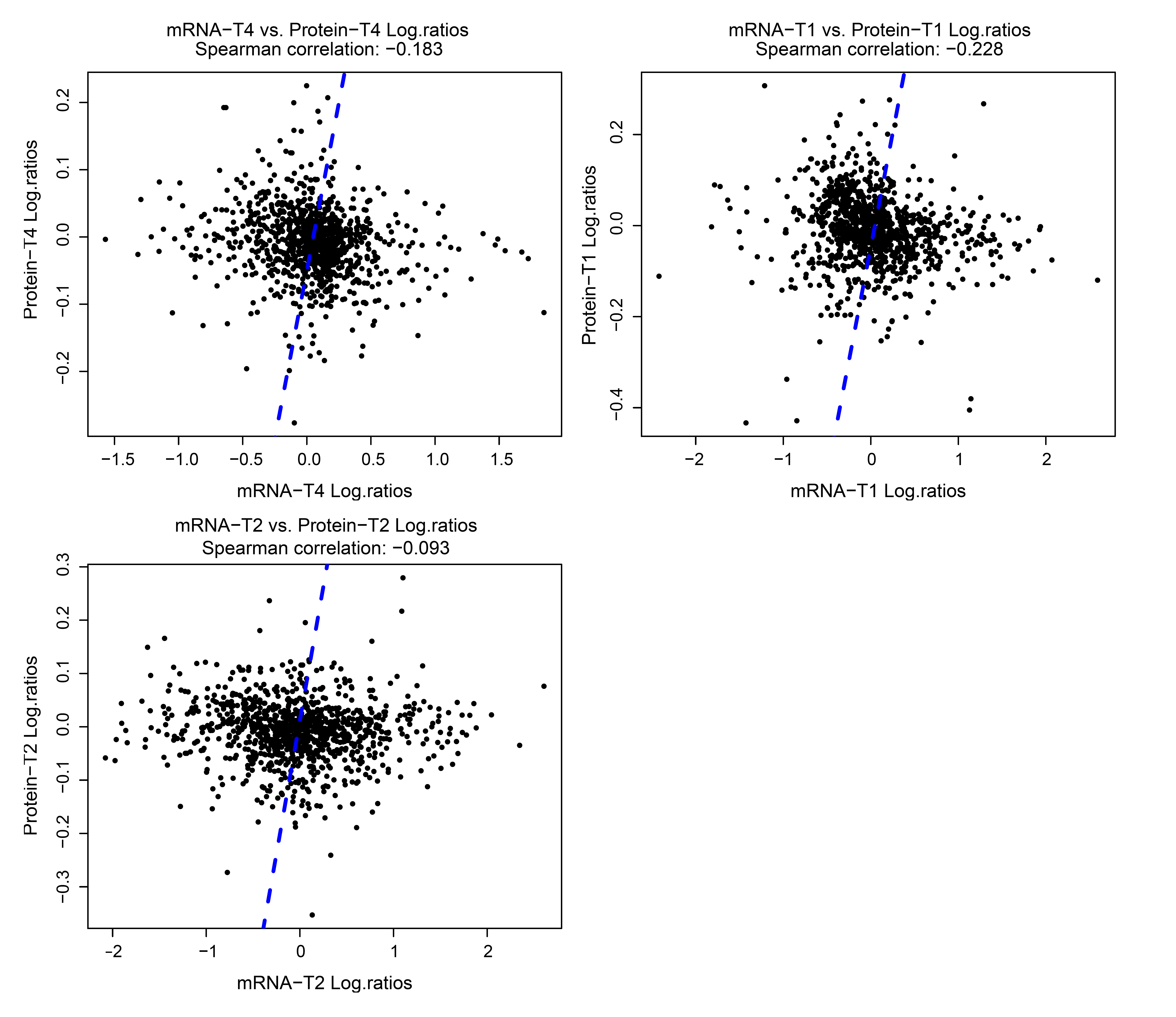


### Figure L: Inverse CDF of the distribution of Pearson Correlation coefficients.

The graph shows the percentage of genes that exceed a given value of the correlation, specified by the x-axis. Less than 40% of the genes in the high-confidence gene set have a Pearson’s correlation coefficient that is positive (>0).


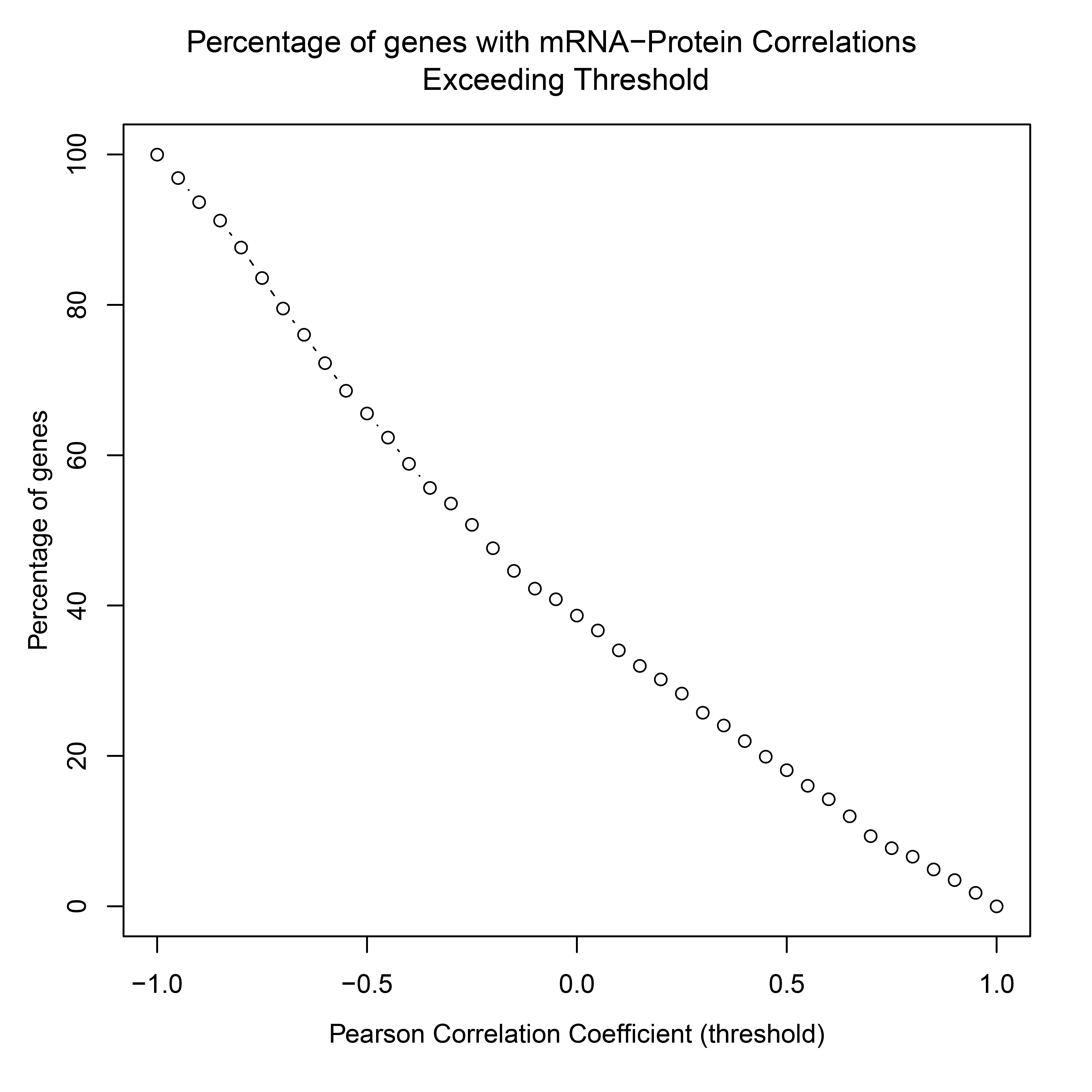


### Figure M: Pathway Concordance GSEA signatures for Log-ratios for concordant pathways.

Heatmap of the concordant pathways, from the Gene Set Enrichment Analysis (GSEA) of pairwise correlation of log-ratios. Similar to Figure 3B, this indicates there is considerable concordance between the expression programs of several key metabolic pathways, such as the Oxygenic Photosynthesis and Calvin-Benson-Bassham Cycle pathways.


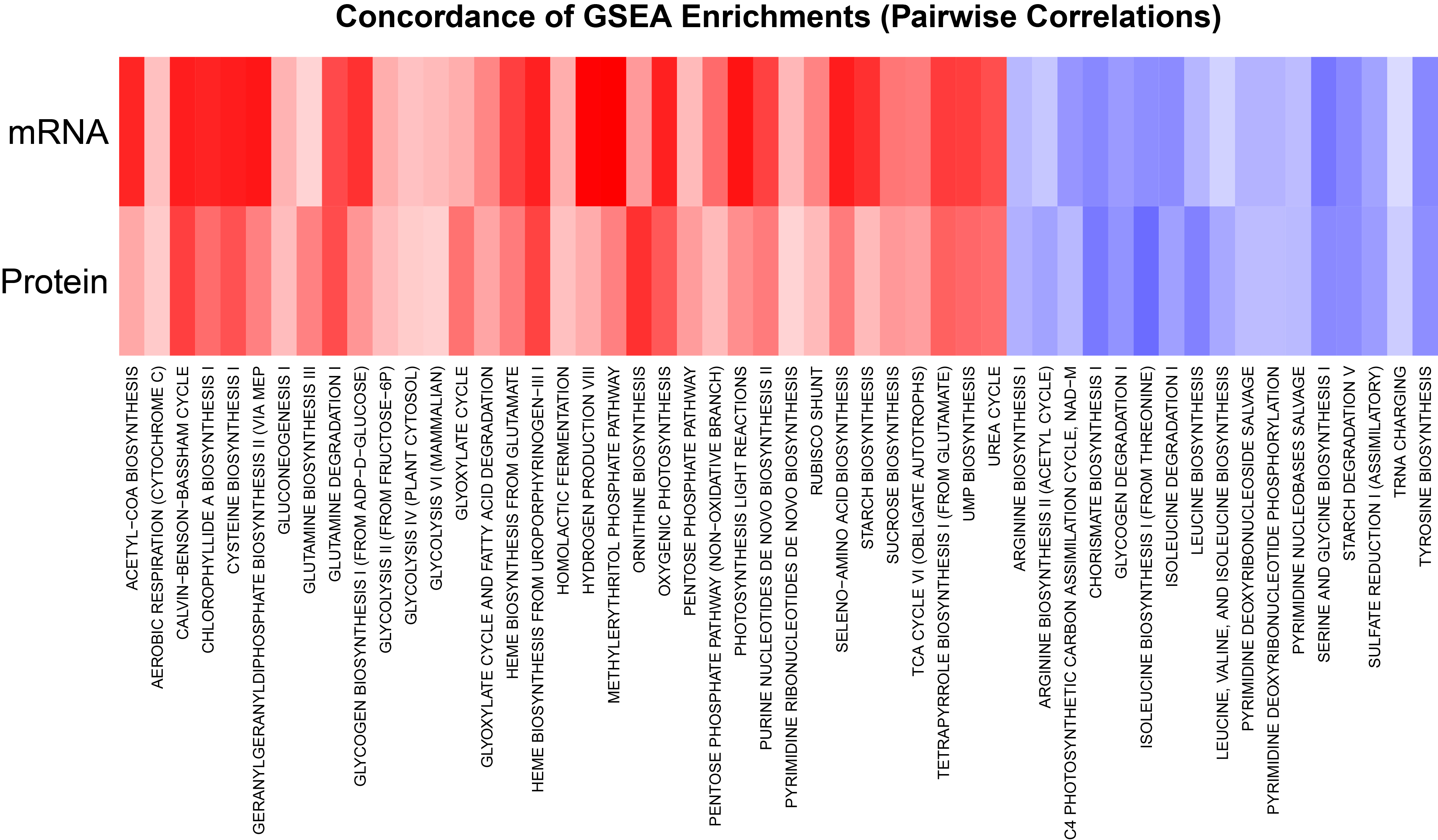


### Figure N: Pathway Concordance between GSEA signatures for Abundances for all pathways


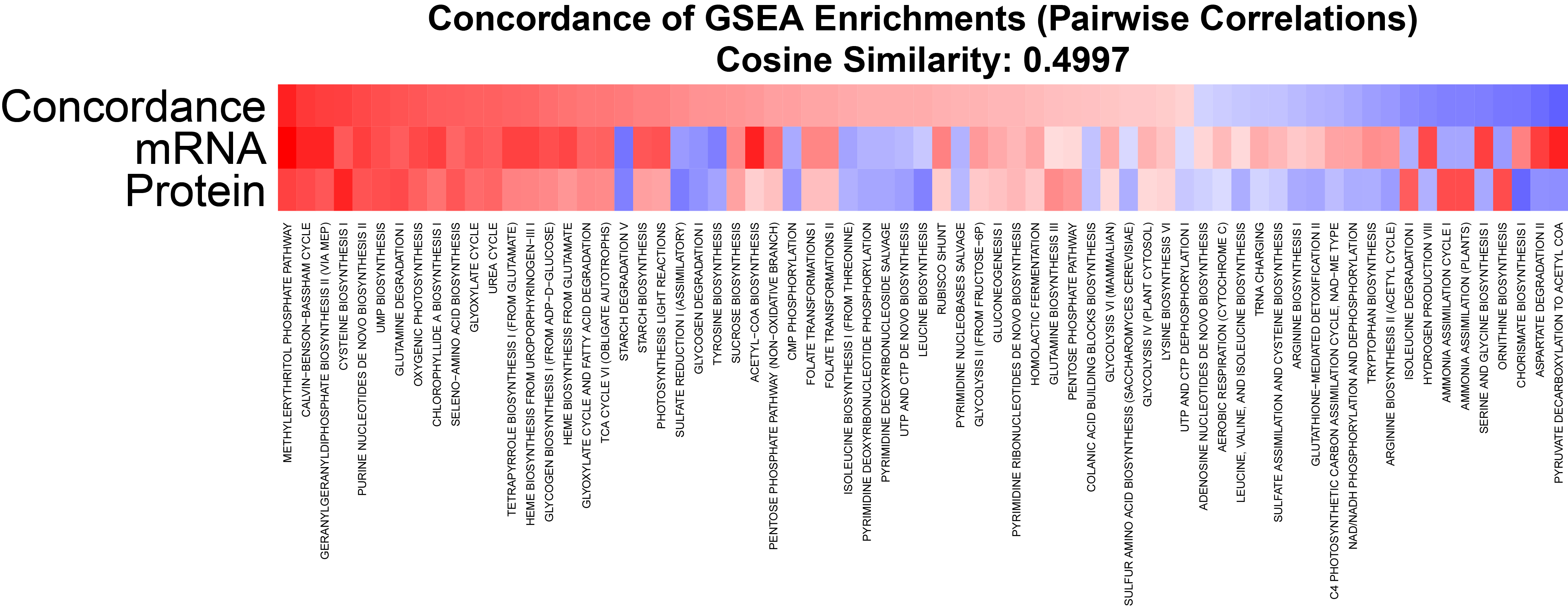


### Figure O: Pathway Concordance between GSEA signatures for Log-Ratios for all pathways


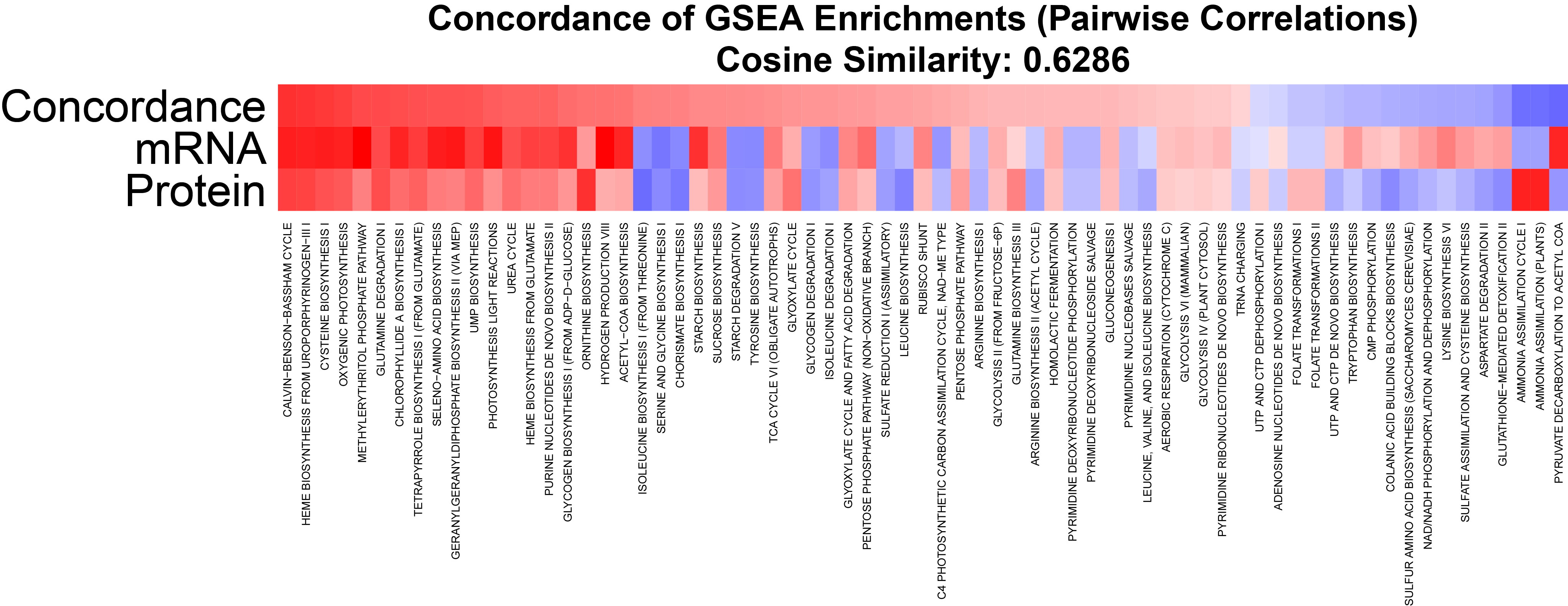


### Figure P: Expression profiles for the Oxygenic Photosynthesis (OP) pathway.

Figure contains the profiles of both clusters and genes that are part of the OP pathway. When a gene is listed, rather than a cluster, this indicates that this gene was the sole member of the OP pathway in a given cluster, and is being included for completeness.


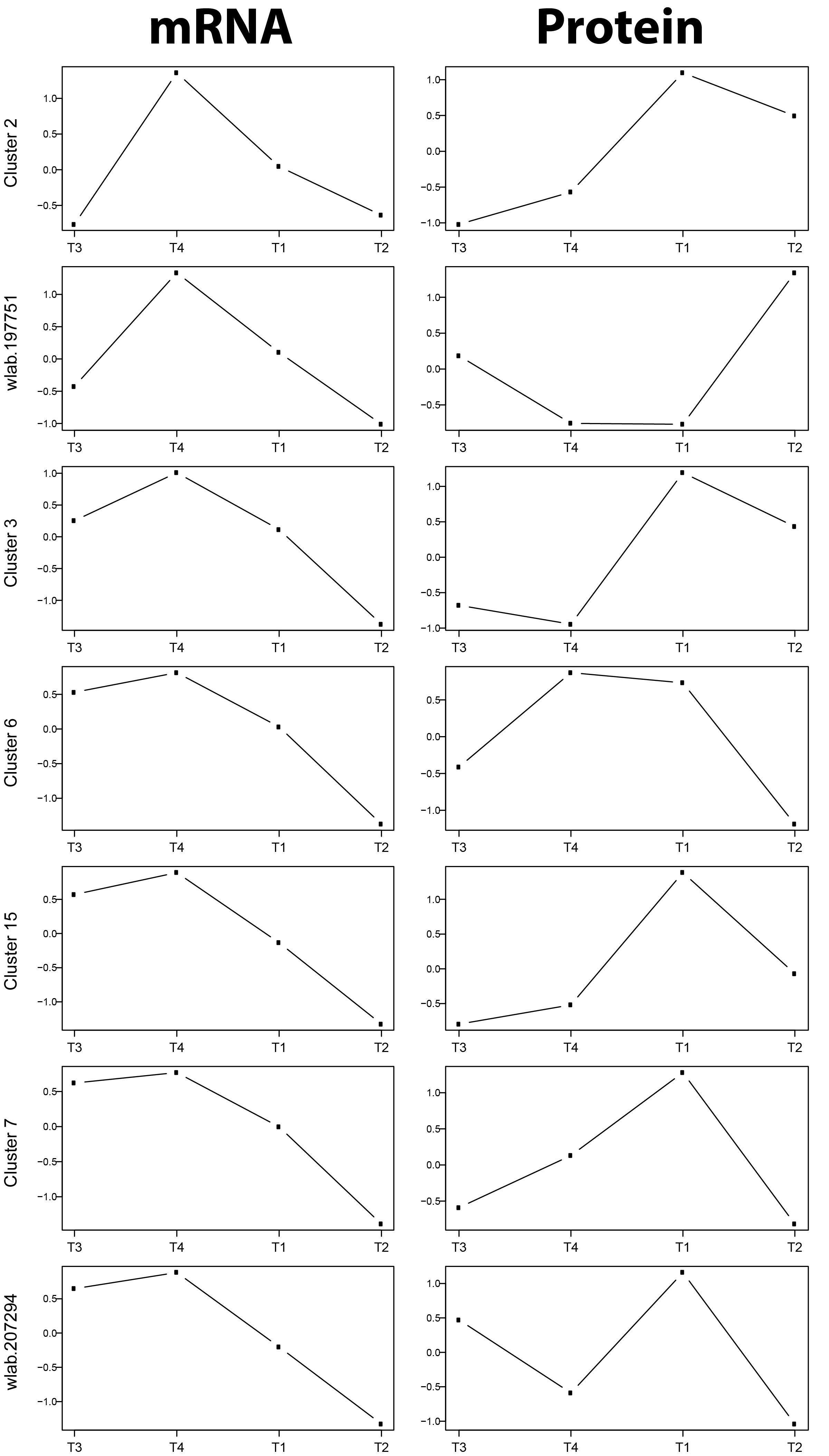


### Figure Q: Partial Correlation Heatmap.

Partial correlation matrix of the most correlated and anti-correlated features that were significantly partially correlated with protein expression in any time points, as indicated by bootstrap testing. As such, this is a superset of those features shown in Figure 5A. The left four columns indicate the Spearman correlation between mRNA expression and the features per time point; the right four columns show the partial correlation of the features with the protein expression, when accounting for their correlation with the mRNA expression.


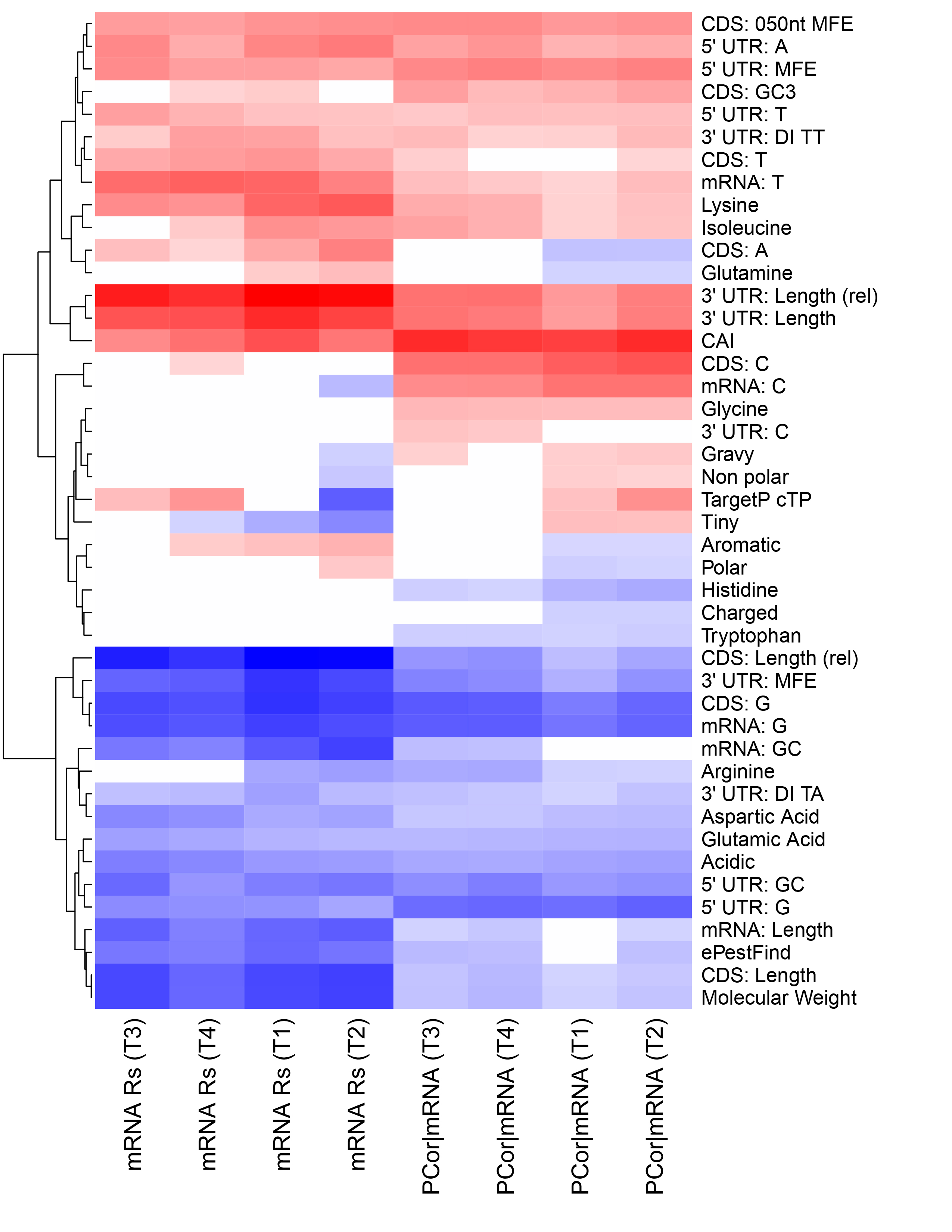


### **Figure R: Gene-wise cross-validation MARS model prediction results.**


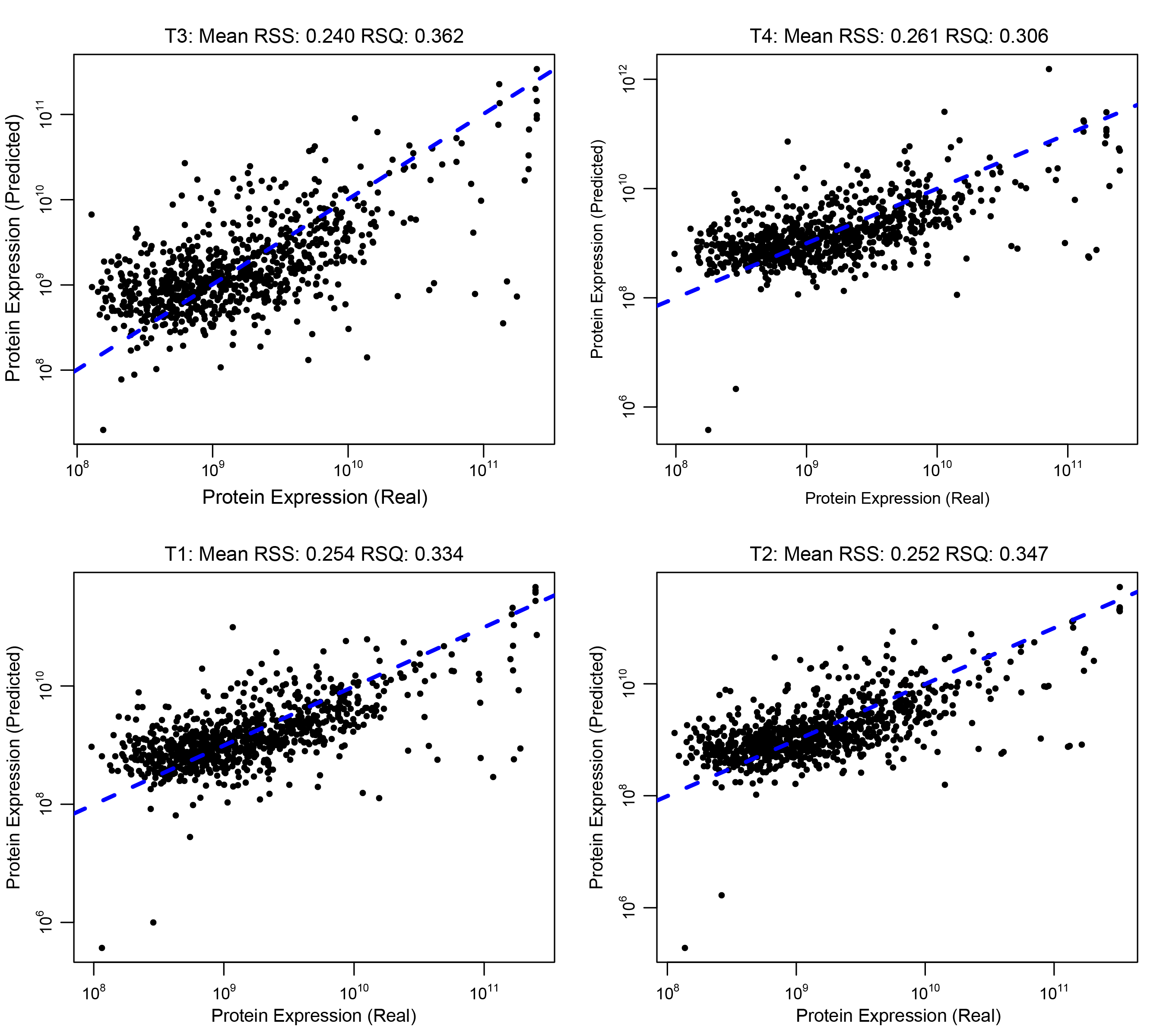


### **Figure S: MARS models when genes in the HPTR set are excluded.**


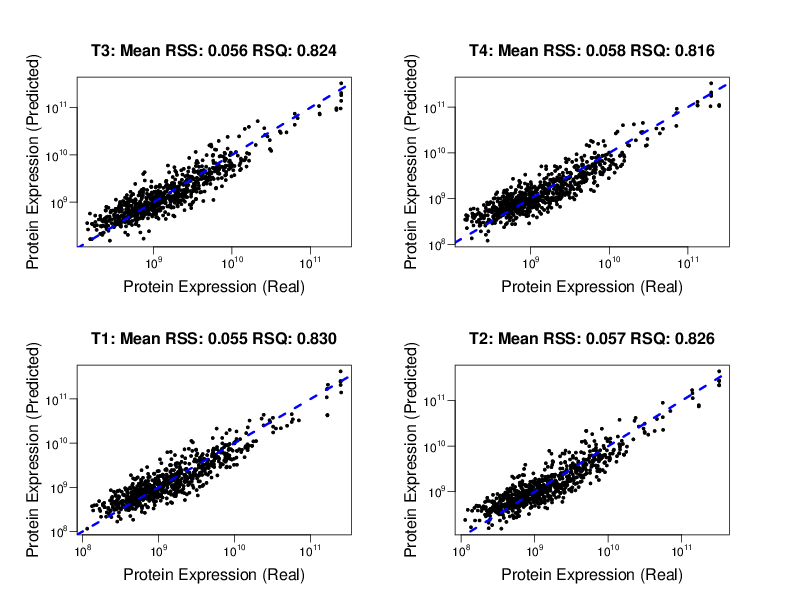


### **Figure T: Cross-validated (10-fold) MARS models when genes in the HPTR set are excluded.**


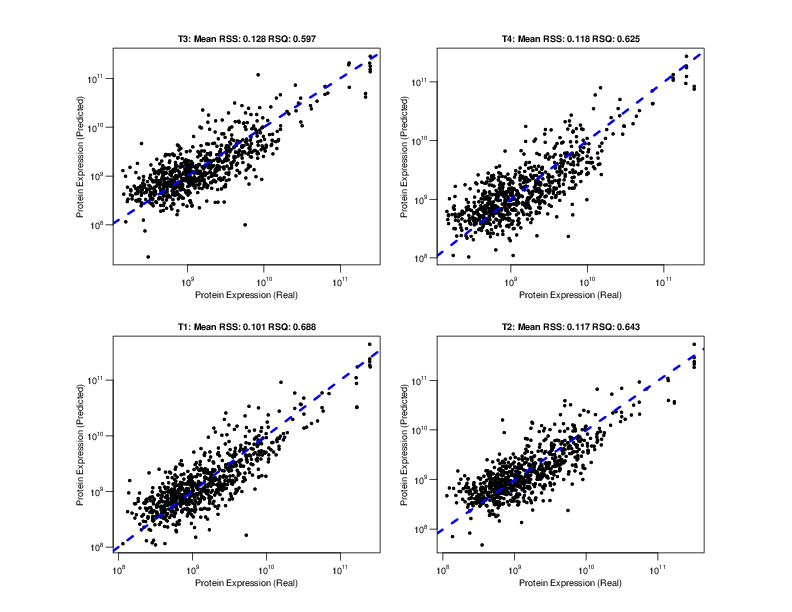


### **Figure U: Distribution of Balanced Success Rates for the binary classifiers generated for the clusters identified.**


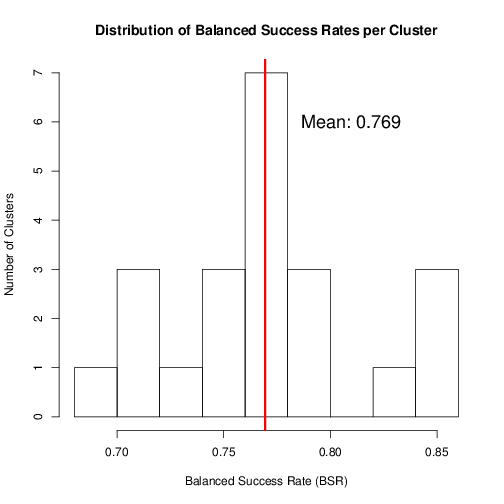


# Supplementary Tables

### Table A: Summary of pathways inferred from PathoLogic for *Micromonas pusilla*.

| Pathways: | 270 |
| --- | --- |
| Enzymatic Reactions: | 1771 |
| Transport Reactions: | 9 |
| Polypeptides: | 9887 |
| Protein Complexes: | 0 |
| Enzymes: | 1957 |
| Transporters: | 35 |
| Compounds: | 1240 |
| Transcription Units: | 0 |
| tRNAs: | 33 |

### Table B: Sequence features used as proxies for mechanisms of post-transcriptional control

| **Features used** | **Method & Explanation** |
| --- | --- |
| Sequence lengths & relative sequence lengths of CDS, mRNA , UTRs (3' and 5') | R(seqinR) [[1](#_ENREF_1)]. Relative sequence lengths are normalized to total length of mRNA |
| Composition of Nucleotides, Dinucleotides, Amino Acids | R(seqinR): residue frequencies are given as normalized by the total number of residues in the respective sequence part. As presented in [[2](#_ENREF_2)], we searched for over/under- represented dinuclotides. |
| G+C content: GC, GC1, GC2, GC3 | R(seqinR): the higher the value, the higher the combined frequency of Guanine and Cytosine.   - Global G+C content = GC - GC1: G+C in the first codon position = GC1, normalized by length; similarly for GC2 and GC3. |
| Amino acid properties | R(seqinR) |
| Codon Bias: CBI (Codon Bias Index), PI, CAI, FOP score, GRAVY score, and AROMATICITY score | Parsed using CodonW [[3](#_ENREF_3), [4](#_ENREF_4)] : the higher the Codon Bias Index, the more biased is the codon usage in the sequence. Highly expressed proteins were taken from the mRNA expression data. |
| Potential causes of Ribosomal attachment in 5' UTR. Upstream AUG and open read frames (AUT w/in frame STOP codon) | Custom R scripts. The five to ten nucleotides surrounding the translation initiation site are thought to influence initiation efficiency [[5](#_ENREF_5)]. |
| Degree of intrinsic unstructuredness of the protein: | - Percent of sequence that is disordered from DisoPred [[6](#_ENREF_6), [7](#_ENREF_7)]: the larger the value for a given protein, the more intrinsically unstructured regions in its sequence (and the less stable it may be). - DisEMBL: coils & hotloops from DisEMBL [[8](#_ENREF_8)]: Disordered proteins tend to be less stable than folded proteins and vice versa. Disorder is measured by loops/coils and “hot loops” (loops with a high degree of mobility) |
| Secondary structure in 5’ or 3’ UTR | RNAfold from the Vienna RNA package [[9](#_ENREF_9)] to predict the folding energy of the sequences. The smaller the energy, the more stable the secondary structures. |
| PEST protein degradation signal | ePESTfind [[10](#_ENREF_10), [11](#_ENREF_11)]: Maximum score |

### Table C: Total number of differentially expressed proteins, relative to previous time point, per KOG class

|  | **Num. differentially Expressed** | | | | **Percent of Class** | | | |
| --- | --- | --- | --- | --- | --- | --- | --- | --- |
| **By KOG Classification:** | **T3vT4** | **T4vT1** | **T1vT2** | **T2vT3** | **T3vT4** | **T4vT1** | **T1vT2** | **T2vT3** |
| **No KOG CLASS** | 213 | 294 | 85 | 78 | 32 | 44 | 13 | 12 |
| **Amino acid transport and metabolism** | 7 | 15 | 1 | 0 | 30 | 65 | 4 | 0 |
| **Carbohydrate transport and metabolism** | 6 | 9 | 3 | 1 | 32 | 47 | 16 | 5 |
| **Cell cycle control, cell division, chromosome partitioning** | 1 | 2 | 0 | 0 | 33 | 67 | 0 | 0 |
| **Cell motility** | 0 | 1 | 0 | 0 | 0 | 100 | 0 | 0 |
| **Cell wall/membrane/envelope biogenesis** | 1 | 2 | 1 | 1 | 20 | 40 | 20 | 20 |
| **Chromatin structure and dynamics** | 2 | 5 | 0 | 1 | 25 | 63 | 0 | 13 |
| **Coenzyme transport and metabolism** | 5 | 3 | 2 | 1 | 45 | 27 | 18 | 9 |
| **Cytoskeleton** | 7 | 11 | 5 | 0 | 30 | 48 | 22 | 0 |
| **Defense mechanisms** | 3 | 3 | 0 | 0 | 50 | 50 | 0 | 0 |
| **Energy production and conversion** | 6 | 9 | 2 | 1 | 33 | 50 | 11 | 6 |
| **Extracellular structures** | 1 | 1 | 0 | 0 | 50 | 50 | 0 | 0 |
| **Function unknown** | 8 | 9 | 4 | 3 | 33 | 38 | 17 | 13 |
| **General function prediction only** | 11 | 23 | 7 | 7 | 23 | 48 | 15 | 15 |
| **Inorganic ion transport and metabolism** | 3 | 6 | 0 | 0 | 33 | 67 | 0 | 0 |
| **Intracellular trafficking, secretion, and vesicular transport** | 3 | 9 | 1 | 1 | 21 | 64 | 7 | 7 |
| **Lipid transport and metabolism** | 2 | 6 | 1 | 3 | 17 | 50 | 8 | 25 |
| **Nuclear structure** | 0 | 2 | 1 | 0 | 0 | 67 | 33 | 0 |
| **Nucleotide transport and metabolism** | 2 | 5 | 0 | 0 | 29 | 71 | 0 | 0 |
| **Posttranslational modification, protein turnover, chaperones** | 14 | 23 | 7 | 6 | 28 | 46 | 14 | 12 |
| **Replication, recombination and repair** | 1 | 2 | 1 | 0 | 25 | 50 | 25 | 0 |
| **RNA processing and modification** | 7 | 7 | 6 | 0 | 35 | 35 | 30 | 0 |
| **Secondary metabolites biosynthesis, transport and catabolism** | 0 | 2 | 0 | 0 | 0 | 100 | 0 | 0 |
| **Signal transduction mechanisms** | 2 | 5 | 0 | 1 | 25 | 63 | 0 | 13 |
| **Transcription** | 4 | 5 | 4 | 1 | 29 | 36 | 29 | 7 |
| **Translation, ribosomal structure and biogenesis** | 29 | 35 | 7 | 1 | 40 | 49 | 10 | 1 |

### Table D: Number of differentially expressed proteins, relative to previous time point.

|  | **Num. differentially Expressed** | | | | **Percent of Class** | | | |
| --- | --- | --- | --- | --- | --- | --- | --- | --- |
|  | **T3vT4** | **T4vT1** | **T1vT2** | **T2vT3** | **T3vT4** | **T4vT1** | **T1vT2** | **T2vT3** |
| **Total up-expressed** | 5 | 458 | 119 | 17 | 1 | 76 | 20 | 3 |
| **Total down-expressed** | 333 | 36 | 19 | 89 | 70 | 8 | 4 | 19 |

### Table E: Listing of enriched metabolic pathways for clusters 3, 6, 7 and 15.

Cluster 2 lacked any enriched pathways, but was enriched in GO terms associated with photosynthesis (see Table 3).

| **Cluster** | **Pathway** | **P-value** |
| --- | --- | --- |
| 3 | chlorophyllide a biosynthesis I | 0.001184 |
|  | methylerythritol phosphate pathway | 0.003255 |
|  | glycogen biosynthesis I (from ADP-D-Glucose) | 0.010652 |
|  | starch biosynthesis | 0.01448 |
|  | superpathway of geranylgeranyldiphosphate biosynthesis II (via MEP) | 0.054712 |
| 6 | superpathay of heme biosynthesis from glutamate | 0.006622 |
|  | chlorophyllide a biosynthesis I | 0.006622 |
|  | Calvin-Benson-Bassham cycle | 0.030375 |
|  | oxygenic photosynthesis | 0.032509 |
| 7 | Calvin-Benson-Bassham cycle | 0.001516 |
|  | sucrose biosynthesis | 0.002164 |
|  | oxygenic photosynthesis | 0.009513 |
|  | gluconeogenesis I | 0.062768 |
|  | glycolysis II (from fructose-6P) | 0.071799 |
|  | homolactic fermentation | 0.076556 |
| 15 | Calvin-Benson-Bassham cycle | 0.000178 |
|  | oxygenic photosynthesis | 0.000755 |
|  | chlorophyllide a biosynthesis I | 0.023698 |
|  | Rubisco shunt | 0.109027 |
|  | gluconeogenesis I | 0.349624 |
|  | glycolysis II (from fructose-6P) | 0.381814 |
|  | homolactic fermentation | 0.397875 |

### Table F: Gene lists for the clusters that contain genes in the Oxygenic Photosynthesis (OP) pathway.

| **Cluster 02:** | **Cluster 03:** | **Cluster 06:** | **Cluster 07:** | **Cluster 12:** | **Cluster 15:** | **Cluster 18:** |
| --- | --- | --- | --- | --- | --- | --- |
| wlab.196263 | wlab.149815 | wlab.152910 | wlab.151452 | wlab.197751 | wlab.151674 | wlab.207294 |
| wlab.196795 | wlab.152551 | wlab.196249 | wlab.152904 |  | wlab.168536 |  |
|  |  | wlab.209350 | wlab.167911 |  | wlab.168896 |  |
|  |  | wlab.223910 | wlab.203563 |  | wlab.202603 |  |
|  |  |  |  |  | wlab.206027 |  |
|  |  |  |  |  | wlab.212926 |  |
|  |  |  |  |  | wlab.223058 |  |
|  |  |  |  |  | wlab.223519 |  |
|  |  |  |  |  |  |  |
| **Non-OP genes below:** | **Non-OP genes below:** | **Non-OP genes below:** | **Non-OP genes below:** | **Non-OP genes below:** | **Non-OP genes below:** | **Non-OP genes below:** |
| wlab.140461 | wlab.140467 | wlab.149511 | wlab.148741 | wlab.148450 | wlab.138205 | wlab.149830 |
| wlab.150370 | wlab.144418 | wlab.149751 | wlab.150723 | wlab.148931 | wlab.146982 | wlab.152248 |
| wlab.151514 | wlab.149361 | wlab.149891 | wlab.150748 | wlab.149908 | wlab.148146 | wlab.155761 |
| wlab.151622 | wlab.149412 | wlab.150427 | wlab.151952 | wlab.150132 | wlab.148493 | wlab.164834 |
| wlab.151882 | wlab.149606 | wlab.150437 | wlab.152307 | wlab.150172 | wlab.150133 | wlab.166257 |
| wlab.152185 | wlab.149625 | wlab.150640 | wlab.153015 | wlab.150233 | wlab.150585 | wlab.168675 |
| wlab.152672 | wlab.149827 | wlab.152809 | wlab.156541 | wlab.153002 | wlab.151210 | wlab.196430 |
| wlab.153070 | wlab.150462 | wlab.153154 | wlab.164683 | wlab.158780 | wlab.151442 | wlab.202249 |
| wlab.153420 | wlab.159260 | wlab.154929 | wlab.169839 | wlab.159525 | wlab.151771 | wlab.203526 |
| wlab.167192 | wlab.159349 | wlab.159654 | wlab.170682 | wlab.168691 | wlab.153410 | wlab.208542 |
| wlab.169470 | wlab.169410 | wlab.165894 | wlab.177337 | wlab.169655 | wlab.154770 | wlab.208981 |
| wlab.169748 | wlab.169599 | wlab.168329 | wlab.196058 | wlab.169678 | wlab.159158 | wlab.209726 |
| wlab.195486 | wlab.170869 | wlab.169215 | wlab.196067 | wlab.195470 | wlab.164658 | wlab.210489 |
| wlab.195867 | wlab.195447 | wlab.171448 | wlab.196869 | wlab.196108 | wlab.168870 | wlab.210984 |
| wlab.196034 | wlab.195982 | wlab.197055 | wlab.196922 | wlab.196346 | wlab.171000 | wlab.211402 |
| wlab.196122 | wlab.196129 | wlab.201371 | wlab.200648 | wlab.199859 | wlab.195781 | wlab.214405 |
| wlab.197542 | wlab.196269 | wlab.202449 | wlab.202094 | wlab.202664 | wlab.195981 | wlab.216258 |
| wlab.201419 | wlab.196310 | wlab.203551 | wlab.202659 | wlab.203689 | wlab.196826 | wlab.222332 |
| wlab.202303 | wlab.197334 | wlab.203673 | wlab.204863 | wlab.205697 | wlab.197895 | wlab.223168 |
| wlab.202632 | wlab.197668 | wlab.205683 | wlab.206356 | wlab.208540 | wlab.198511 | wlab.223521 |
| wlab.203198 | wlab.198099 | wlab.206841 | wlab.207011 | wlab.208956 | wlab.202460 | wlab.223577 |
| wlab.204333 | wlab.198722 | wlab.207676 | wlab.208680 | wlab.209677 | wlab.202583 | wlab.223615 |
| wlab.205756 | wlab.200129 | wlab.207834 | wlab.209072 | wlab.210142 | wlab.202613 | wlab.224046 |
| wlab.206360 | wlab.201445 | wlab.208769 | wlab.209223 | wlab.210235 | wlab.202856 | wlab.230044 |
| wlab.206818 | wlab.201940 | wlab.208948 | wlab.210828 | wlab.210537 | wlab.204439 |  |
| wlab.206836 | wlab.202455 | wlab.209566 | wlab.210949 | wlab.211807 | wlab.206887 |  |
| wlab.206969 | wlab.202564 | wlab.209597 | wlab.210956 | wlab.212027 | wlab.206917 |  |
| wlab.206983 | wlab.202640 | wlab.210154 | wlab.211027 | wlab.212724 | wlab.207598 |  |
| wlab.206995 | wlab.202694 | wlab.210175 | wlab.211075 | wlab.214523 | wlab.207804 |  |
| wlab.207809 | wlab.203234 | wlab.210425 | wlab.212363 | wlab.221184 | wlab.208481 |  |
| wlab.208050 | wlab.206042 | wlab.210543 | wlab.212505 | wlab.222896 | wlab.208494 |  |
| wlab.208476 | wlab.206096 | wlab.210817 | wlab.214664 | wlab.223402 | wlab.208579 |  |
| wlab.208592 | wlab.206172 | wlab.212941 | wlab.220020 | wlab.223545 | wlab.208653 |  |
| wlab.208804 | wlab.206405 | wlab.214826 |  | wlab.223582 | wlab.208995 |  |
| wlab.209012 | wlab.206756 | wlab.215394 |  | wlab.223878 | wlab.209133 |  |
| wlab.209092 | wlab.206922 | wlab.218919 |  |  | wlab.209140 |  |
| wlab.209116 | wlab.207017 | wlab.221790 |  |  | wlab.209157 |  |
| wlab.209409 | wlab.207069 | wlab.223539 |  |  | wlab.209338 |  |
| wlab.209525 | wlab.207378 | wlab.223549 |  |  | wlab.209503 |  |
| wlab.210001 | wlab.207469 | wlab.223779 |  |  | wlab.209539 |  |
| wlab.210741 | wlab.207610 | wlab.223800 |  |  | wlab.209632 |  |
| wlab.211602 | wlab.207928 | wlab.223898 |  |  | wlab.209838 |  |
| wlab.211870 | wlab.208376 | wlab.223962 |  |  | wlab.210008 |  |
| wlab.211871 | wlab.208740 | wlab.223981 |  |  | wlab.210814 |  |
| wlab.212048 | wlab.209125 | wlab.223996 |  |  | wlab.211082 |  |
| wlab.212051 | wlab.209633 | wlab.224066 |  |  | wlab.211358 |  |
| wlab.212337 | wlab.209634 |  |  |  | wlab.211445 |  |
| wlab.212424 | wlab.209708 |  |  |  | wlab.211603 |  |
| wlab.212770 | wlab.209881 |  |  |  | wlab.211803 |  |
| wlab.213330 | wlab.210184 |  |  |  | wlab.212321 |  |
| wlab.215140 | wlab.210563 |  |  |  | wlab.212562 |  |
| wlab.220013 | wlab.210574 |  |  |  | wlab.212663 |  |
| wlab.220022 | wlab.210638 |  |  |  | wlab.212771 |  |
| wlab.220046 | wlab.210659 |  |  |  | wlab.214560 |  |
| wlab.221670 | wlab.210729 |  |  |  | wlab.215361 |  |
| wlab.222320 | wlab.210891 |  |  |  | wlab.217284 |  |
| wlab.222331 | wlab.211077 |  |  |  | wlab.223213 |  |
| wlab.223838 | wlab.211098 |  |  |  | wlab.223588 |  |
| wlab.223850 | wlab.211273 |  |  |  | wlab.223598 |  |
| wlab.223944 | wlab.211434 |  |  |  | wlab.223628 |  |
|  | wlab.211455 |  |  |  | wlab.223630 |  |
|  | wlab.211848 |  |  |  | wlab.223738 |  |
|  | wlab.211964 |  |  |  | wlab.223890 |  |
|  | wlab.212201 |  |  |  | wlab.223902 |  |
|  | wlab.212262 |  |  |  |  |  |
|  | wlab.212628 |  |  |  |  |  |
|  | wlab.212657 |  |  |  |  |  |
|  | wlab.212757 |  |  |  |  |  |
|  | wlab.219985 |  |  |  |  |  |
|  | wlab.220955 |  |  |  |  |  |
|  | wlab.221004 |  |  |  |  |  |
|  | wlab.222457 |  |  |  |  |  |
|  | wlab.223609 |  |  |  |  |  |
|  | wlab.223625 |  |  |  |  |  |
|  | wlab.223634 |  |  |  |  |  |
|  | wlab.223643 |  |  |  |  |  |
|  | wlab.223782 |  |  |  |  |  |
|  | wlab.223787 |  |  |  |  |  |
|  | wlab.223798 |  |  |  |  |  |
|  | wlab.223857 |  |  |  |  |  |
|  | wlab.224048 |  |  |  |  |  |

### Table G: Listing of GO term enrichments for clusters enriched with Oxygenic Photosynthesis clusters (2, 3, 7, 15).

| **Cluster** | **Term** | **Annotated** | **Significant** | **P-value** |
| --- | --- | --- | --- | --- |
| 2 | photosynthesis | 69 | 18 | 4.50E-21 |
|  | photosynthesis, light reaction | 26 | 10 | 9.50E-14 |
|  | photosynthesis, light harvesting | 19 | 8 | 1.50E-11 |
| 3 | photosynthesis | 69 | 10 | 1.90E-07 |
|  | chlorophyll biosynthetic process | 10 | 5 | 3.30E-07 |
|  | translation | 253 | 16 | 3.80E-06 |
|  | isoprenoid biosynthetic process | 17 | 5 | 7.50E-06 |
| 7 | isoprenoid biosynthetic process | 17 | 2 | 0.0076 |
| 15 | photosynthesis | 69 | 7 | 3.10E-05 |
|  | photosynthesis, light harvesting | 19 | 3 | 0.00198 |
|  | carbohydrate metabolic process | 163 | 7 | 0.00587 |
|  | branched-chain amino acid biosynthetic p... | 10 | 2 | 0.00765 |
|  | chlorophyll biosynthetic process | 10 | 2 | 0.00765 |

### Table H: List of MARS sequence features used (50+% of samples).

| **Amino Acids/Classes** | | **mRNA/CDS sequence Features** |
| --- | --- | --- |
| - Alanine - Cysteine - Glutamic Acid - Glutamine - Glycine - Leucine - Lysine - Methionine - Phenylalanine - Proline - Tyrosine - Valine | - Acidic - Aromatic - Basic - Charged - Polar - Tiny | - CAI - CDS Adenine - CDS Guanine - mRNA Cytosine proportion - mRNA Thymine proportion - mRNA GC1^++^ |
| **UTR Features:**   - 3’ UTR MFE - 3’ UTR Dinucleotide TT - 3’ UTR GC Enrichment - 3’ UTR length | | - 5’ UTR Adenine - 5’ UTR Tyrosine Proportion - 5’ UTR GC Enrichment |
| **Miscellaneous:**   - DisEMBL Coils - Isoelectric Point - TargetP (chloroplast) | | ^++^G/C enrichment of n_th_ codon position, e.g. GC1 is the G/C enrichment of the 1^st^ position of the codon bases |

### Table I: GO Enrichments of the HPTR genes

| **Genes Under-Estimated by MARS Model (n=88):** | | | | | | |
| --- | --- | --- | --- | --- | --- | --- |
| **GO Branch** | **GO Annotation** | **GO Description** | **# Genes Annotated** | **# Found in Set** | **Number Expected** | **P-value** |
| BP | GO:0006334 | nucleosome assembly | 18 | 4 | 0.35 | 0.00468 |
| MF | GO:0051287 | NADP binding | 28 | 5 | 0.30 | 0.00018 |
| MF | GO:0050661 | NAD binding | 23 | 4 | 0.36 | 0.00041 |
| MF | GO:0016620 | oxidoreductase activity, acting on the a... | 15 | 3 | 0.19 | 0.00083 |
| MF | GO:0046933 | proton-transporting ATP synthase activit... | 12 | 2 | 0.15 | 0.00992 |
| MF | GO:0046961 | proton-transporting ATPase activity, rot... | 12 | 2 | 0.15 | 0.00992 |
| CC | GO:0000786 | nucleosome | 17 | 3 | 0.39 | 0.0061 |

### Table J: Features that are significantly different for the Oxygenic Photosynthesis clusters (clusters 6, 7 and 15).

| **Cluster** | **Greater/Enriched** | **Smaller/Depleted** | |
| --- | --- | --- | --- |
| **6** | - 5’ UTR MFE - 5’ UTR uORF - TargetP (chloroplast) - mRNA Cytosine (%) - Cysteine - Valine | - 5’ UTR Guanine (%) - CDS Adenine (%) - Tyrosine | |
| **7** | - 5’ UTR Cytosine (%) - mRNA GC enrichment - TargetP (chloroplast) - Glycine - Serine - Valine - Tiny | - 5’ UTR Adenine (%) | |
| **15** | - CAI - TargetP (chloroplast) - mRNA Cytosine (%) - Alanine - Serine | - Histidine - Lysine - Leucine | - Aliphatic - Basic - Polar |

# Supplementary materials and methods

## Preparation of Proteomics Data

### Peptide Analysis by Liquid Chromatography Tandem Mass Spectrometry (LC-MS/MS).

Two chromatography approaches were utilized to maximize peptide separation and increase proteome coverage when analyzed by LC-MS/MS. First, peptides from the global, soluble, and insoluble lysis fractions were fractionated off-line (LC not coupled to the mass spectrometry instrument) using reversed-phase, high pH chromatography as previously described [[12](#_ENREF_12)]. The second approach utilized a 2D-LC on-line (coupled) separation [[13](#_ENREF_13)].

### Peptide sequence identification

Mass spectrometric data were subjected to sequence analysis using the SEQUEST algorithm [[14](#_ENREF_14)], ThermoElectron, San Jose, Ca. version 27 (rev. 12)) which compares MS/MS spectra to a sequence repository, specifically the collection of all gene models derived from the genome by JGI. Briefly, SEQUEST input files were created using in-house parent isotope correction algorithm DeconMSn [[15](#_ENREF_15)] (omics.pnl.gov), a parent mass tolerance of +/-3Da was employed to capture remaining de-isotoping errors, a static modification was applied to all Cysteine residues to reflect alkylation with Iodoacetamide (+57.0215 Da), and no proteolytic enzyme was specified. The output from this analysis was then rescored using the MSGF spectral probability algorithm [[16](#_ENREF_16)]. A subset of the full dataset was analyzed using a target-decoy approach, whereby all sequences are combined with their reverse compliment in a single search file, allowing false discovery rates (FDR) to be assessed at given quality level cutoffs [[17](#_ENREF_17)]. It was observed that an MSGF spectral probability E-value of less than or equal to 1E-10 provided an FDR of <1%, and this value was used to filter subsequent data.

## Expression-based cluster classification

While the MARS models provided a global view of the processes governing protein expression, we sought to explore the possibility of identifying features that were predictive for individual clusters. To accomplish this, we generated discriminative models for each cluster using linear models that sought parsimonious sets of features using an elastic net regularization [[18](#_ENREF_18)] to generate binary class predictors for each gene module. Briefly, elastic net regression uses a regularization strategy to enforce model sparsity which employs a user-specified mixture of L_1_ (Lasso) and L_2_ (Ridge) penalties, with the goal of allowing the combined penalties to balance out their respective shortcomings. For completeness, classifier testing was also performed using regression trees, naïve Bayesian, and support vector classifiers via the RWeka CRAN package [[19](#_ENREF_19)]. However, as our goal with the binary-classification analysis was to identify features that were discriminative for each cluster, GLMNET was a more appropriate choice due to the feature selection inherent to the method.

As we sought to identify features that were both discriminative, as well as having putative explanatory power for the protein abundances, we constrained these models to only consider features that were also used by the global MARS models. Additionally, to avoid a well-known classification bias due to imbalanced sizes of the dichotomous classes when generating the binary predictors, class membership was balanced using convex pseudo-data (CPD henceforth. However, to evaluate the accuracy of these discriminative models, we excluded the pseudo-data samples from the evaluation. As this yielded an unbalanced data set, we used the balanced success rate (BSR henceforth) [[20](#_ENREF_20)] as our evaluation metric. Briefly, the BSR is the average of the success rates (percent correct) for each class. While we present the definition in the main text, we present it again below:

$$BSR=\frac{{SR}_{1}+{SR}_{2}}{2}$$

where SR_1_ and SR_2_ are the success rates for classes 1 and 2, where the success rate is defined as the fraction of true positives (TP) correctly classified, i.e. $SR=TP/\left( TP+FN \right)$.

As the generation of CPD contains a random component, we performed 100 experiments for each binary classifier, and used the average BSR from these experiments as the final score for a given gene cluster. The average of these average BSR’s for each of the binary classifiers was 0.77 (*p*<0.0002; Figure U).

As a final step, we examined the features that had been selected in a majority of the experiments that were performed for a given gene cluster. Using Wilcoxon’s non-parametric rank sum test, we compared the distribution from the cluster genes for a given feature with that from the remainder of the genes in the high-confidence gene set. In Table J, we list features that were statistically different (greater or smaller) for clusters 6, 7 and 15. Unsurprisingly, all three of these clusters were enriched with genes that were predicted by TargetP to be localized to the chloroplast, however, this was the only feature which all three clusters shared. In contrast, cluster 15 was the only cluster that contained genes with larger CAI scores than the rest of the gene set. While the cluster 15 genes have a greater CAI score, it is unclear why they would also exhibit a protein expression profile that appears to be delayed by one time point. While the cluster 15 genes have a greater proportions of alanine, as well as lower proportions of histidine, lysine and leucine, along with the amino acid classes that these belong to (aliphatic, basic and polar amino acids); it is not clear how these residues may influence protein translation or degradation.

While there is no clear explanation for the temporal expression differences that were observed for the cluster 15 genes, RNA secondary structure may potentially explain the differences that are observed for the cluster 7 genes. While not definitive, cluster 7 was the only cluster that exhibited GC enrichment. While this enrichment was only observed in the mRNA sequences as a whole, the 5’ UTRs of the cluster 7 genes also had a greater proportion of cytosine than the rest of the high-confidence gene set – which may be a reflection of a non-statistically significant increase in GC enrichment. As GC-enrichment has been shown to be associated with increased RNA secondary structure that has been shown to decrease ribosomal efficiency [[21](#_ENREF_21)], both of these features may provide evidence for RNA structure within the 5’ UTRs that influences the translation of the cluster 7 genes.

Stronger evidence for RNA secondary structure influencing translation is provided by cluster 6, which was the only cluster that was enriched for genes having both greater minimum free energy (MFE) as well as a smaller portion of guanine in the 5’UTR. While the greater number of uORFs present in the 5’ UTRs of these genes would lend support for a slower rate of translation, the higher MFE indicates decreased structure in the 5’ UTR for the cluster 6 genes. As greater RNA structure in 5’ UTR has been associated with decreased translation rates [[21](#_ENREF_21)], this decreased structure may indicate an increased translation rate that may explain the tight correlation between the mRNA and protein expression profiles that was observed for the genes in cluster 6. Further evidence for the decrease 5’ UTR structure may be provided by the decreased proportion of guanine in the cluster 6 genes, which may indicate a lower GC-enrichment for these genes (and a corresponding decrease in structure). Thus, the differing temporal dynamics that were initially observed between the genes in cluster 6 and 7 may be partially explained by this evidence for differing levels of secondary structure within the 5’ UTRS of the two clusters.

**Supplementary References**

1. Charif D, Lobry JR. SeqinR 1.0-2: a contributed package to the R project for statistical computing devoted to biological sequences retrieval and analysis. Structural approaches to sequence evolution: Molecules, netorks, populations. 2007.

2. Karlin S, Cardon LR. Computational DNA sequence analysis. Annual review of microbiology. 1994;48:619-54. Epub 1994/01/01. doi: 10.1146/annurev.mi.48.100194.003155. PubMed PMID: 7826021.

3. Sharp PM, Li WH. The codon Adaptation Index--a measure of directional synonymous codon usage bias, and its potential applications. Nucleic acids research. 1987;15(3):1281-95. Epub 1987/02/11. PubMed PMID: 3547335; PubMed Central PMCID: PMC340524.

4. Peden JF. Analysis of codon usage. PhD Thesis: University of Nottingham, UK.; 1999.

5. Kozak M. An analysis of 5'-noncoding sequences from 699 vertebrate messenger RNAs. Nucleic acids research. 1987;15(20):8125-48. Epub 1987/10/26. PubMed PMID: 3313277; PubMed Central PMCID: PMC306349.

6. Ward JJ, Sodhi JS, McGuffin LJ, Buxton BF, Jones DT. Prediction and functional analysis of native disorder in proteins from the three kingdoms of life. Journal of molecular biology. 2004;337(3):635-45. Epub 2004/03/17. doi: 10.1016/j.jmb.2004.02.002. PubMed PMID: 15019783.

7. Ward JJ, McGuffin LJ, Bryson K, Buxton BF, Jones DT. The DISOPRED server for the prediction of protein disorder. Bioinformatics. 2004;20(13):2138-9. Epub 2004/03/27. doi: 10.1093/bioinformatics/bth195. PubMed PMID: 15044227.

8. Linding R, Jensen LJ, Diella F, Bork P, Gibson TJ, Russell RB. Protein disorder prediction: implications for structural proteomics. Structure. 2003;11(11):1453-9. Epub 2003/11/08. PubMed PMID: 14604535.

9. Gruber AR, Lorenz R, Bernhart SH, Neubock R, Hofacker IL. The Vienna RNA websuite. Nucleic acids research. 2008;36(Web Server issue):W70-4. Epub 2008/04/22. doi: 10.1093/nar/gkn188. PubMed PMID: 18424795; PubMed Central PMCID: PMC2447809.

10. Olson SA. EMBOSS opens up sequence analysis. European Molecular Biology Open Software Suite. Briefings in bioinformatics. 2002;3(1):87-91. Epub 2002/05/11. PubMed PMID: 12002227.

11. Rice P, Longden I, Bleasby A. EMBOSS: the European Molecular Biology Open Software Suite. Trends Genet. 2000;16(6):276-7. Epub 2000/05/29. doi: S0168-9525(00)02024-2 [pii]. PubMed PMID: 10827456.

12. Wang Y, Yang F, Gritsenko MA, Wang Y, Clauss T, Liu T, et al. Reversed-phase chromatography with multiple fraction concatenation strategy for proteome profiling of human MCF10A cells. Proteomics. 2011;11(10):2019-26. Epub 2011/04/19. doi: 10.1002/pmic.201000722. PubMed PMID: 21500348; PubMed Central PMCID: PMC3120047.

13. Robidart J, Callister SJ, Song P, Nicora CD, Wheat CG, Girguis PR. Characterizing Microbial Community and Geochemical Dynamics at Hydrothermal Vents Using Osmotically Driven Continuous Fluid Samplers. Environmental science & technology. 2013;47(9):4399-407. doi: 10.1021/es3037302.

14. Eng JK, McCormack AL, Yates JR. An approach to correlate tandem mass spectral data of peptides with amino acid sequences in a protein database. J Am Soc Mass Spectrom. 1994;5(11):976-89. Epub 1994/11/01. doi: 10.1016/1044-0305(94)80016-2. PubMed PMID: 24226387.

15. Mayampurath AM, Jaitly N, Purvine SO, Monroe ME, Auberry KJ, Adkins JN, et al. DeconMSn: a software tool for accurate parent ion monoisotopic mass determination for tandem mass spectra. Bioinformatics. 2008;24(7):1021-3. Epub 2008/02/29. doi: 10.1093/bioinformatics/btn063. PubMed PMID: 18304935; PubMed Central PMCID: PMC2720627.

16. Kim S, Gupta N, Pevzner PA. Spectral probabilities and generating functions of tandem mass spectra: a strike against decoy databases. Journal of proteome research. 2008;7(8):3354-63. Epub 2008/07/04. doi: 10.1021/pr8001244. PubMed PMID: 18597511; PubMed Central PMCID: PMC2689316.

17. Elias JE, Gygi SP. Target-decoy search strategy for increased confidence in large-scale protein identifications by mass spectrometry. Nat Methods. 2007;4(3):207-14. Epub 2007/03/01. doi: 10.1038/nmeth1019. PubMed PMID: 17327847.

18. Friedman J, Hastie T, Tibshirani R. Regularization Paths for Generalized Linear Models via Coordinate Descent. Journal of statistical software. 2010;33(1):1-22. Epub 2010/09/03. PubMed PMID: 20808728; PubMed Central PMCID: PMC2929880.

19. Hornik K, Buchta C, Zeileis A. Open-source machine learning: R meets Weka. Comput Stat. 2009;24(2):225-32. doi: 10.1007/s00180-008-0119-7.

20. Ben-Hur A, Weston J. A user's guide to support vector machines. Methods Mol Biol. 2010;609:223-39. Epub 2010/03/12. doi: 10.1007/978-1-60327-241-4_13. PubMed PMID: 20221922.

21. Schmidt MW, Houseman A, Ivanov AR, Wolf DA. Comparative proteomic and transcriptomic profiling of the fission yeast Schizosaccharomyces pombe. Molecular systems biology. 2007;3:79. Epub 2007/02/15. doi: 10.1038/msb4100117. PubMed PMID: 17299416; PubMed Central PMCID: PMC1828747.
